# Supplementary figures and images for: FOXP1 orchestrates neurogenesis in human cortical basal radial glial cells
Source: PLoS Biol. 2023 Aug 4;21(8):e3001852. doi: 10.1371/journal.pbio.3001852 (PMC10431666; doi:10.1371/journal.pbio.3001852)

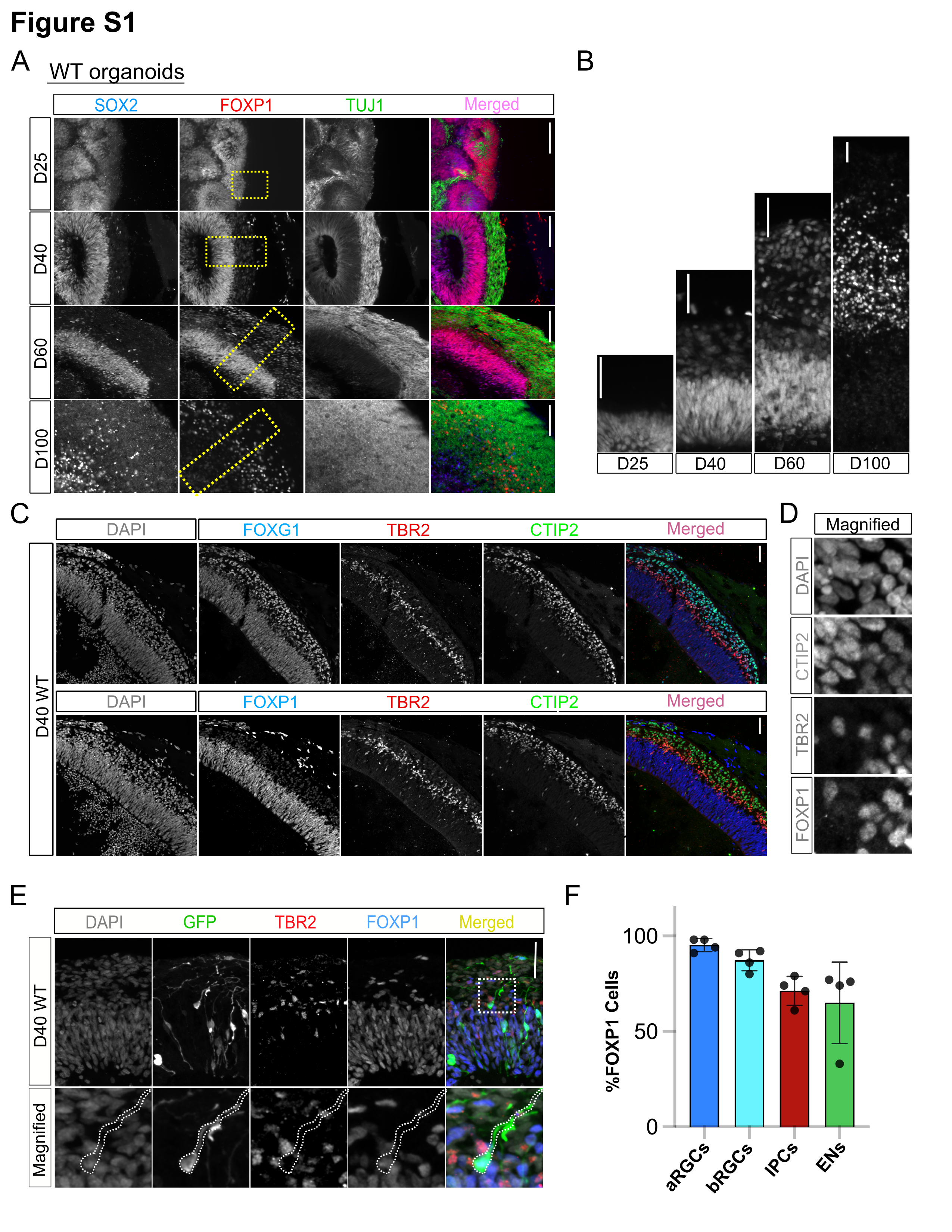

Supplement: S1 Fig — (A) Organoid immunostaining at D25, D40, D60, and D100 showing expression of SOX2, TUJ1, and FOXP1. (B) ROI selected from panel (A) showing FOXP1 expression. (C) Overlap between cell type markers and FOXP1. FOXG1 marks DTL cells, TBR2 marks IPCs, and CTIP2 marks ENs. (D) Magnified images from panel (C) showing the overlap among cell type markers. (E) Detection of “ad-EGFP”-infected FOXP1+ bRGCs based on morphology, location, and absence of TBR2 expression. (F) Quantification of FOXP1+ aRGCs, bRGCs, IPCs, and ENs. Scale bar = 100 μM for panels (A) and (C), 50 μM for panel S1B and S1E Fig. n = 4 cortical structures used from WT organoids. The numerical values that were used to generate the graph can be found in S1 Data. Ad-EGFP, adenovirus-expressing GFP; aRGC, apical radial glial cell; bRGC, basal radial glial cell; DTL, dorsal telencephalic lineage; EN, excitatory neuron; FOXP1, Forkhead Box P1; IPC, intermediate progenitor cell; ROI, region of interest; WT, wild type. (TIFF) [file pbio.3001852.s001.tiff]

Figure S2

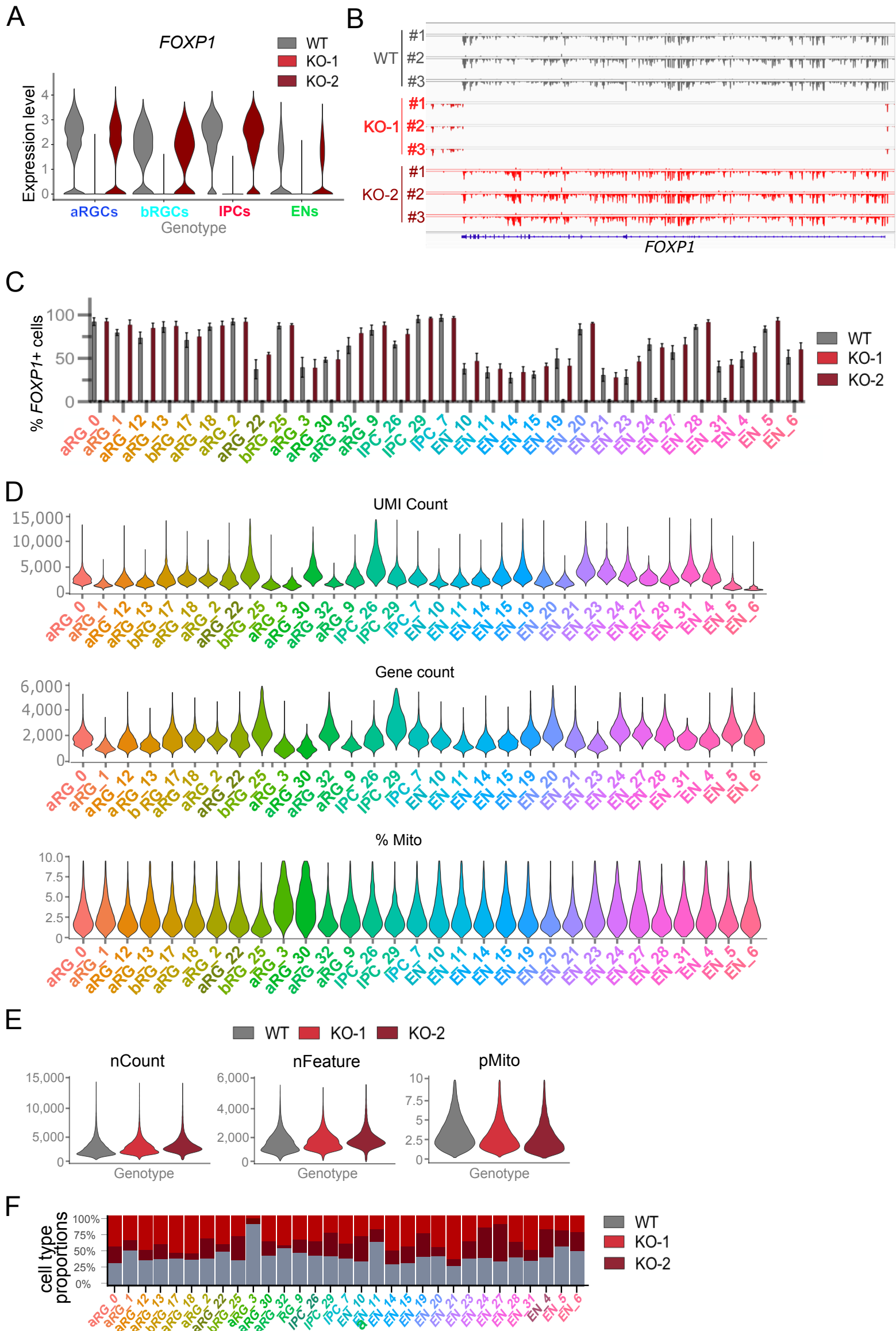

Supplement: S2 Fig — (A) FOXP1 expression level in each of the pseudobulked cell types, aRGCs, bRGCs, IPCs, and ENs per genotype. (B) Genome track files at the FOXP1 gene showing the pileup of the sequencing reads in this region of the genome for each genotype. (C) Percentage of FOXP1+ cells in individual clusters. (D) UMI count, number of detected genes, and percentage mitochondria gene in each cluster. (E) UMI count, number of detected genes, and percentage mitochondrial genes in each genotype. aRGC, apical radial glial cell; bRGC, basal radial glial cell; EN, excitatory neuron; FOXP1, Forkhead Box P1; IPC, intermediate progenitor cell; snRNA-seq, single-nuclei RNA-sequencing. (PDF) [file pbio.3001852.s002.pdf]

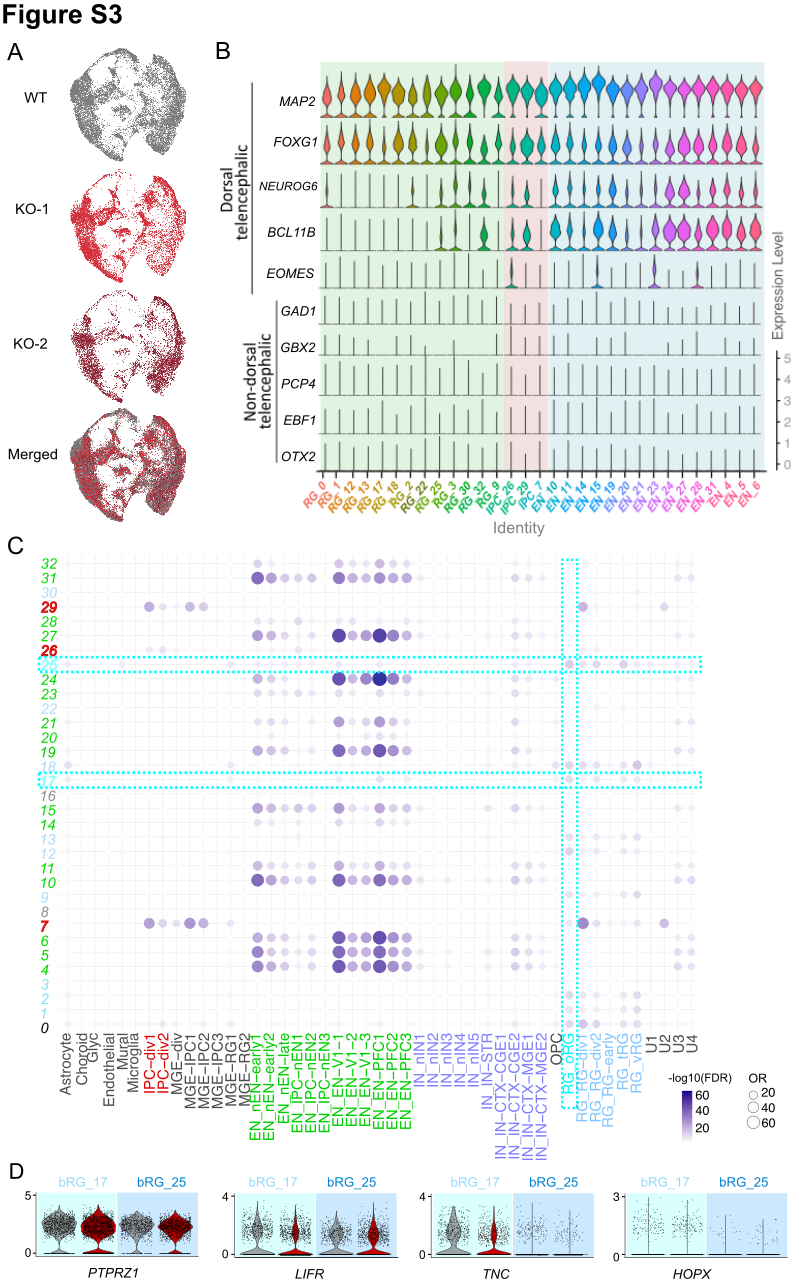

Supplement: S3 Fig — (A) Feature plots showing distribution of cells across different genotypes. (B) Violin plots showing gene expression of markers representing the DTL versus other brain regions. (C) Dot plot showing gene expression correlation between our brain organoid dataset and a human fetal cortex scRNA-seq dataset from the second trimester of gestation [24]. Dotted lines in blue indicate bRGC cell clusters. (D) Violin plots showing the expression level of bRGC-marker genes [5] that are differentially regulated between WT and KO in each bRGC subcluster. Y-axis represents log10(CPM) values. bRGC, basal radial glial cell; DTL, dorsal telencephalic lineage; KO, knockout; OR, odds ratio; scRNA-seq, single-cell RNA-sequencing; WT, wild type. (PNG) [file pbio.3001852.s003.png]

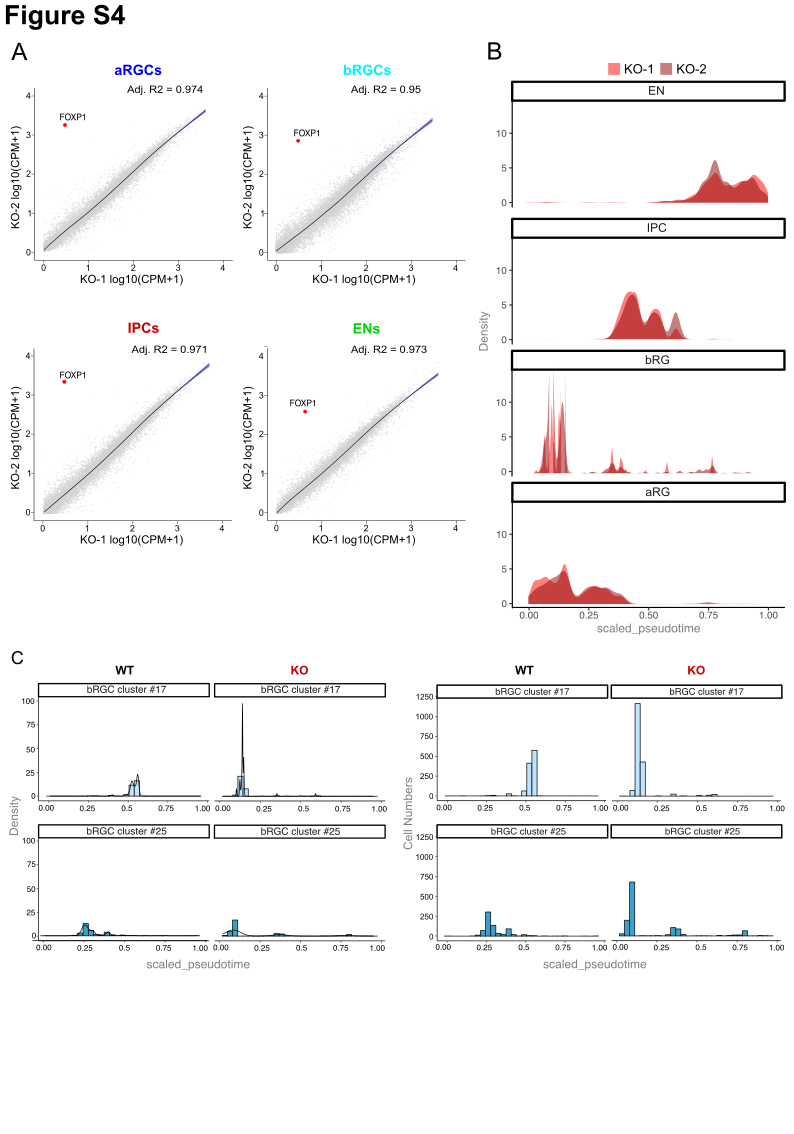

Supplement: S4 Fig — (A) Correlation of DEGs between KO-1 and KO-2 organoids in all cells as well as each of the pseudobulked cell types, aRGC, bRGC, IPC, and ENs. (B) Density plots showing the number of pseudobulked cells (aRGC, bRGC, IPC, and EN) of KO-1 and KO-2 organoids separately. aRGC, apical radial glial cell; bRGC, basal radial glial cell; EN, excitatory neuron; DEG, differentially expressed gene; IPC, intermediate progenitor cell; KO, knockout. (PNG) [file pbio.3001852.s004.png]

**Figure S5**

**A**

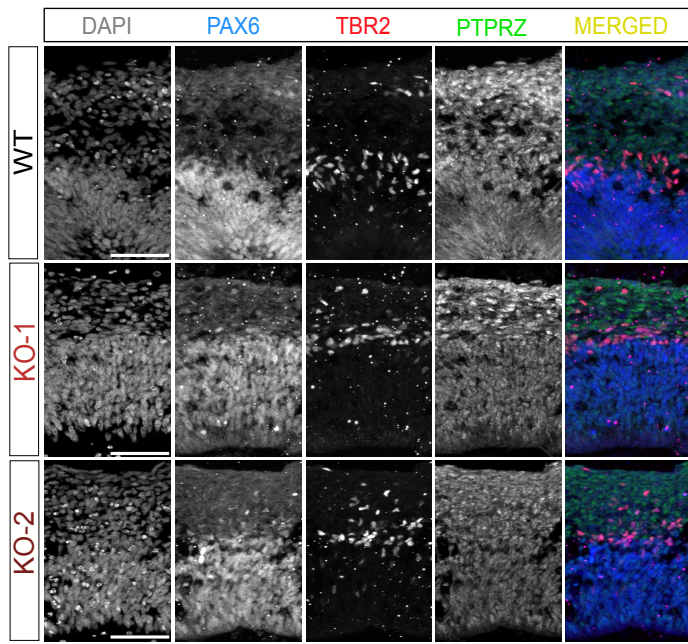

**B**

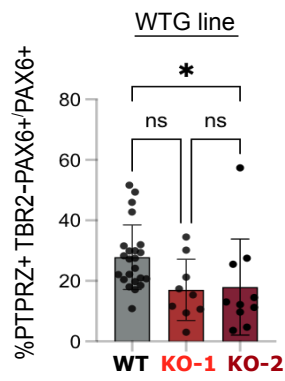

**C**

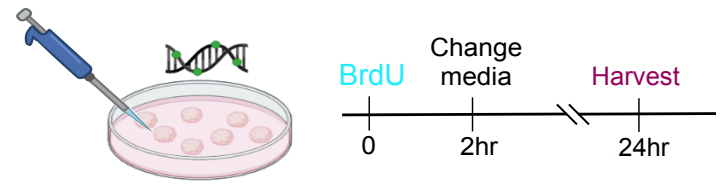

**D**

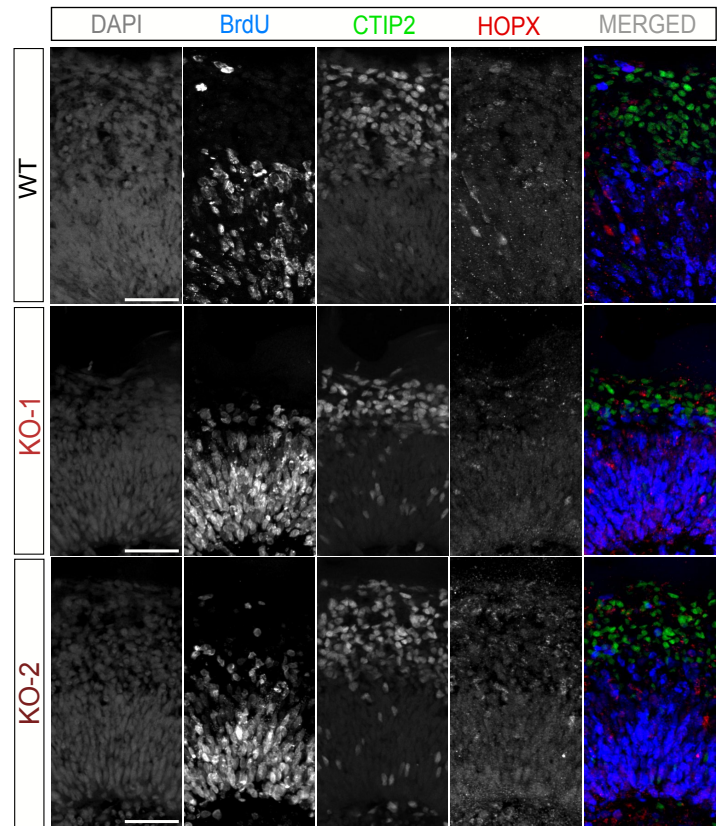

**E**

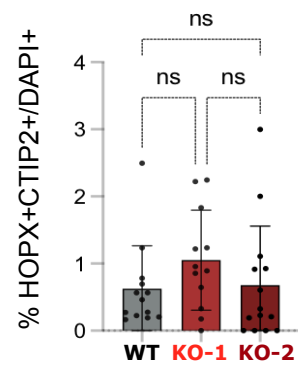

Supplement: S5 Fig — (A) Immunostaining showing PAX6, TBR2, and the bRGC marker PTPRZ in week 6 organoids. Scale bar = 50 μM (B) Quantification of bRGCs using PTPRZ+TBR2− bRGCs. (C) Schematic showing the experimental design of the BrdU treatment at week 6. Created with BioRender.com. (D) Immunostaining showing CTIP2, HOPX, BrdU, and DAPI in week 6 organoids. Scale bar = 50 μM (E) Quantification of neurogenic bRGCs, which are CTIP2+ cells located next to HOPX+ cells. These cells may represent neurons born directly from bRGC to EN. For all quantifications, n = 3–8 organoids per sample per genotype was used. In each organoid, 3–9 cortical structures with clear lamination patterns were examined. Data are represented in bar graphs as mean ± STD with individual data as dots; n.s. = not statistically significant, *p < 0.05, Kruskal–Wallis ANOVA test with Dunn’s multiple comparisons test as a post hoc. The numerical values that were used to generate the graphs can be found in S1 Data. BrdU, 5-bromo-2-deoxyuridine; bRGC, basal radial glial cell; EN, excitatory neuron. (PDF) [file pbio.3001852.s005.pdf]

Figure S6

A

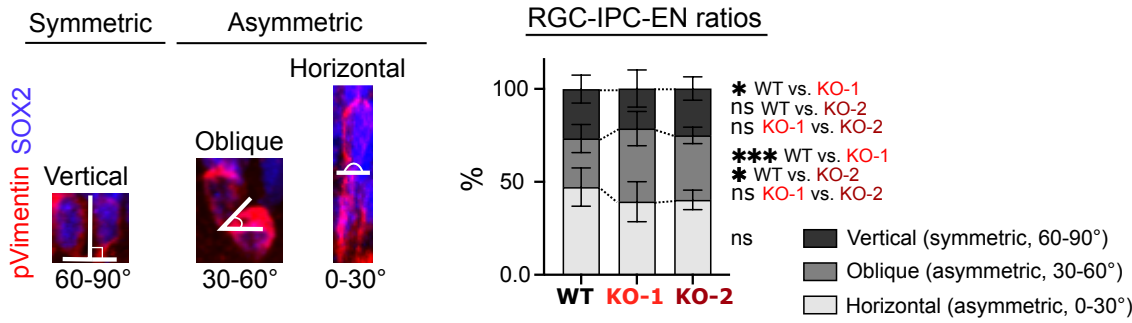

B

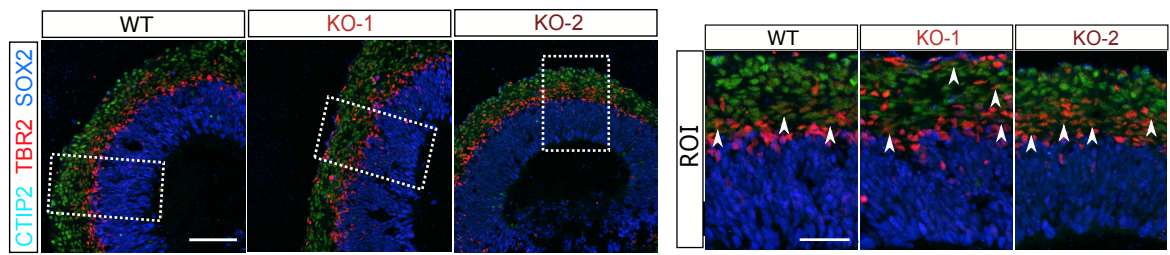

C

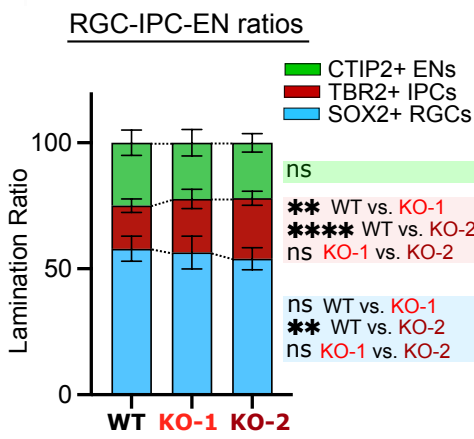

D

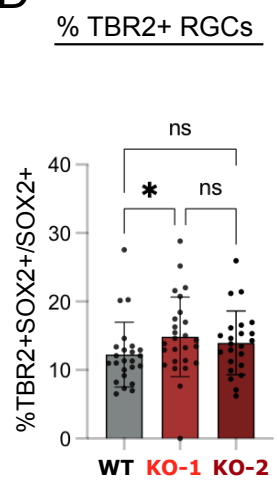

E

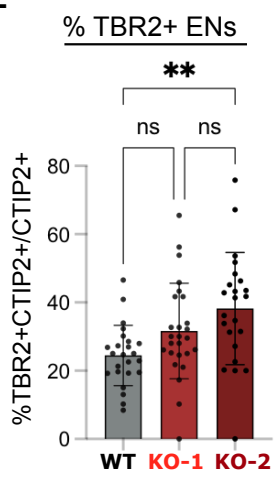

F

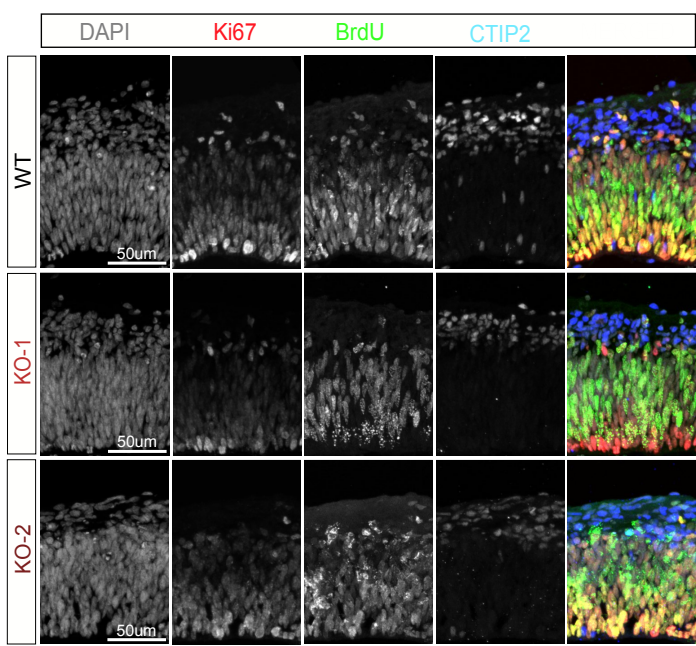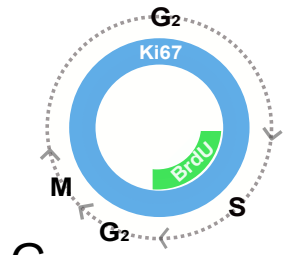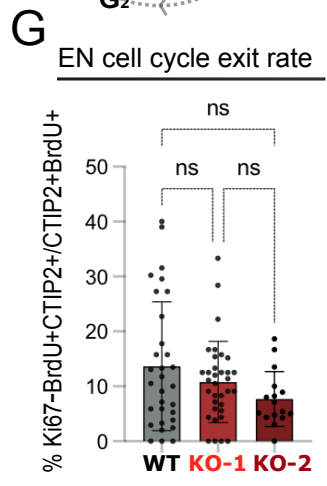

Supplement: S6 Fig — (A) Representative images of symmetric and asymmetric division (left) and quantification of the divisions across WT, KO-1 and KO-2. (B) Representative images showing changes observed in layering with the loss of FOXP1 (left) and examples of ROIs selected from left panel (right). SOX2 expression marks RGCs, TBR2 marks IPCs and CTIP2 marks ENs. Scale bar = 100 μM for the left images and 50 μM for the ROI images on the right. (C) Quantification of RGCs, IPCs, and ENs. (D) Quantification of SOX2+ RGCs that express IPC marker TBR2. (E) Quantification of CTIP2+ ENs that express IPC marker TBR2. (F) Representative images of BrdU-treated brain organoids sectioned and stained for Ki67, BrdU, and CTIP2 expression. Scale bar = 50 μM (G) The difference between BrdU and Ki67 staining showing cell cycle exit rate in the CTIP2+ postmitotic neurons. For all quantifications, n = 3–8 organoids per sample per genotype was used. In each organoid, 3–9 cortical structures with clear lamination patterns were examined. Data are represented in bar graphs as mean ± STD with individual data as dots; n.s. means p > 0.05, *p < 0.05, **p < 0.01, and ****p < 0.0001. Mixed-effects analysis multiple comparisons test with Tukey’s multiple comparisons test as a post hoc was used for panel (C). Kruskal–Wallis ANOVA test with Dunn’s multiple comparisons test as a post hoc was used for S6D Fig and S6E Fig. The numerical values that were used to generate the graphs can be found in S1 Data. BrdU, 5-bromo-2-deoxyuridine; EN, excitatory neuron; FOXP1, Forkhead Box P1; IPC, intermediate progenitor cell; KO, knockout; RGC, radial glial cell; ROI, region of interest; WT, wild type. (PDF) [file pbio.3001852.s006.pdf]

Figure S7

A

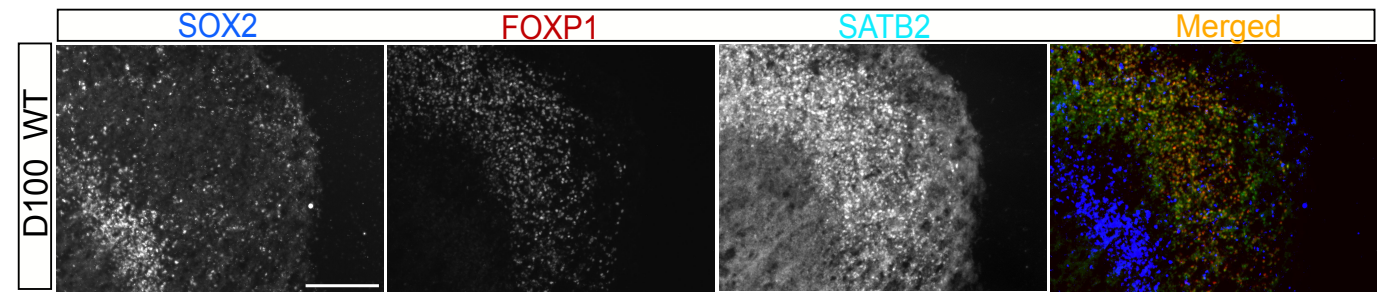

B

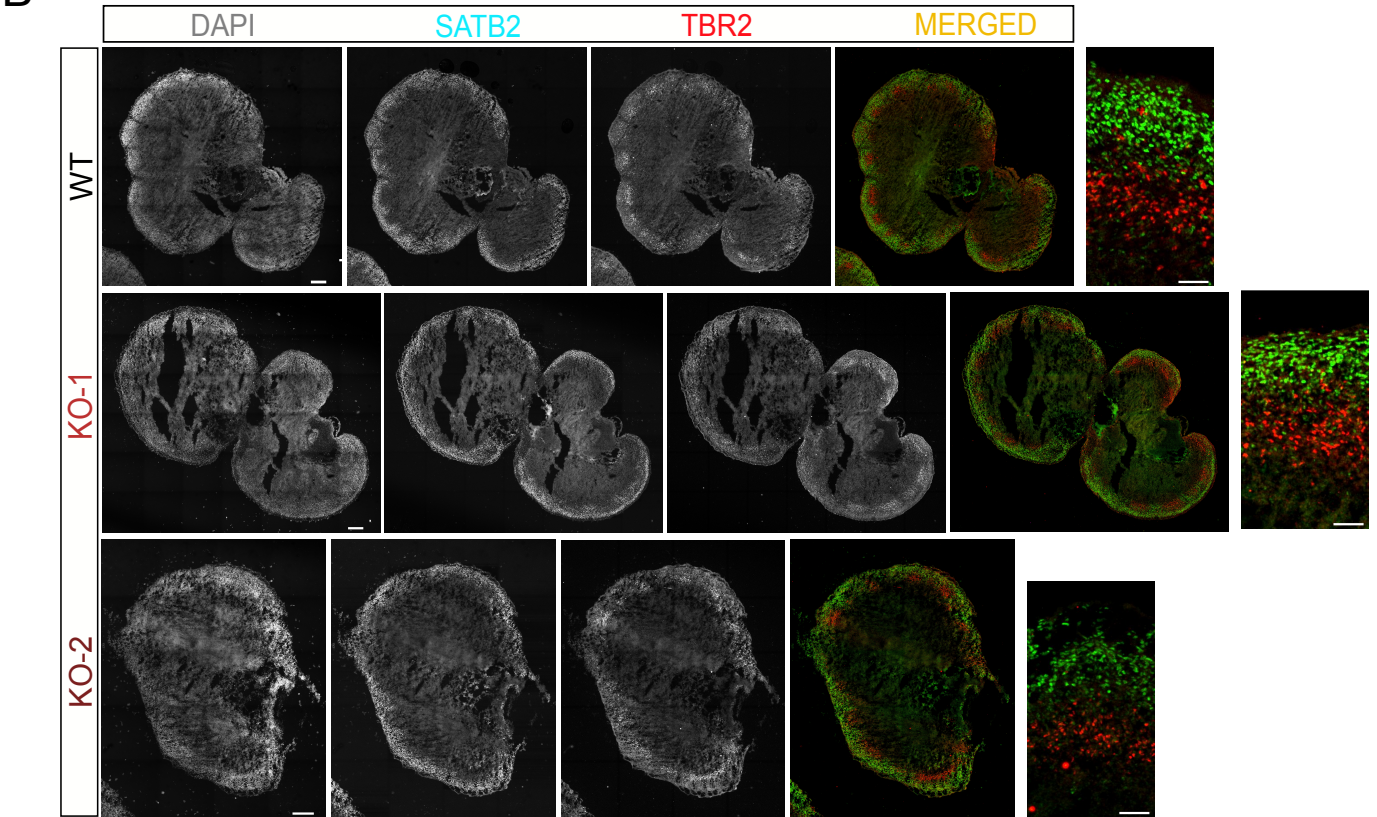

C

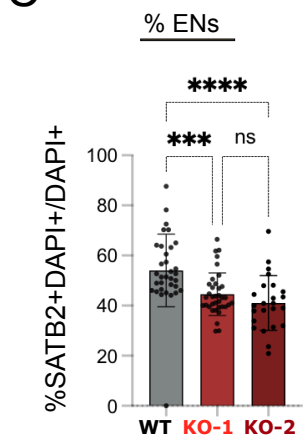

D

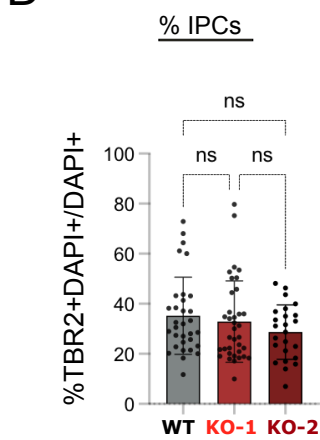

E

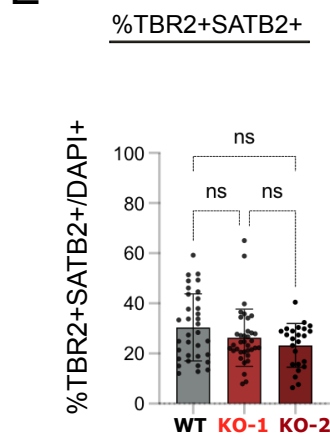

F

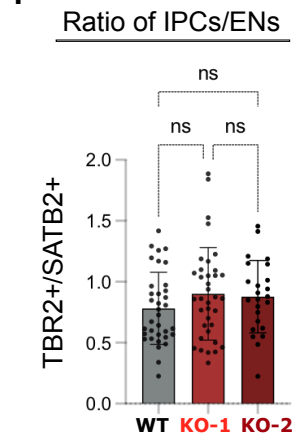

Supplement: S7 Fig — (A) Immunostaining images of D100 organoids showing expression of SOX2 for RGCs, SATB2 for ENs, and FOXP1 in a representative WT organoid. (B) Immunostaining showing IPCs (TBR2+) and ENs (SATB2+) at D100. (C–F) Immunostaining results showing percentage of RGCs, IPCs, and ENs at D100 normalized to all DAPI+ cells. For all quantifications, n = 3–8 organoids per sample per genotype was used. In each organoid, 3–9 cortical structures with clear lamination patterns were examined. Data are represented in bar graphs as mean ± STD with individual data as dots; n.s. means p > 0.05, ***p <0.001, and ****p <0.0001 Kruskal–Wallis ANOVA test with Dunn’s multiple comparisons test as a post hoc was used. Scale bar = 100 μM for S7A Fig. Scale bar = 300 μM for S7B Fig on the left and 100 μM for ROI selected images on the right. The numerical values that were used to generate the graphs can be found in S1 Data. EN, excitatory neuron; FOXP1, Forkhead Box P1; IPC, intermediate progenitor cell; RGC, radial glial cell; ROI, region of interest; WT, wild type. (PDF) [file pbio.3001852.s007.pdf]

Figure S8

A

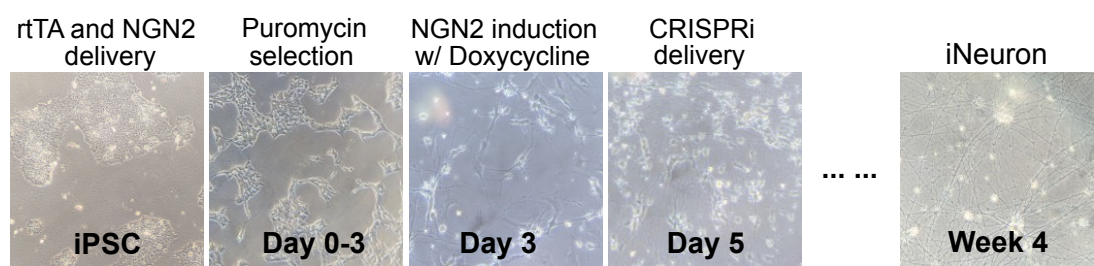

B

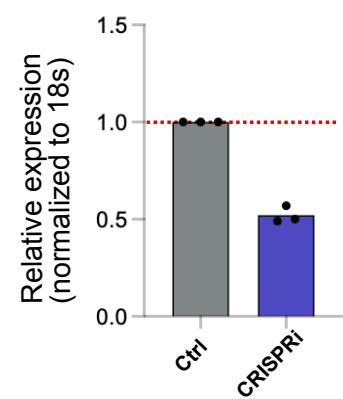

C

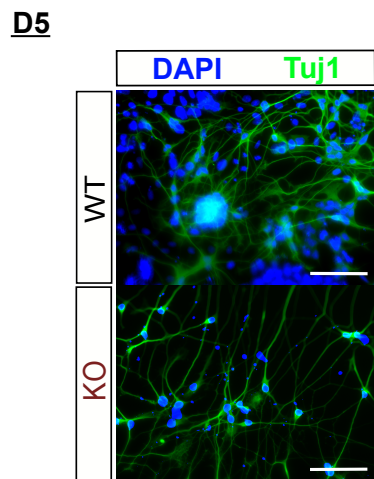

D

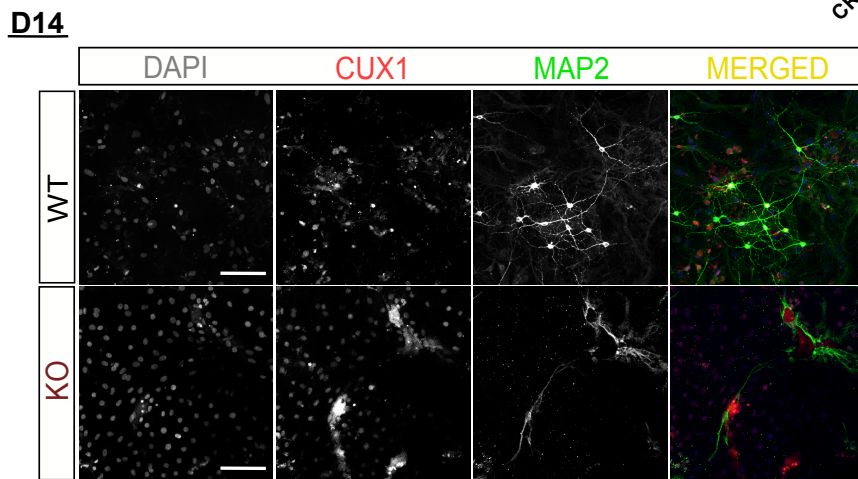

E

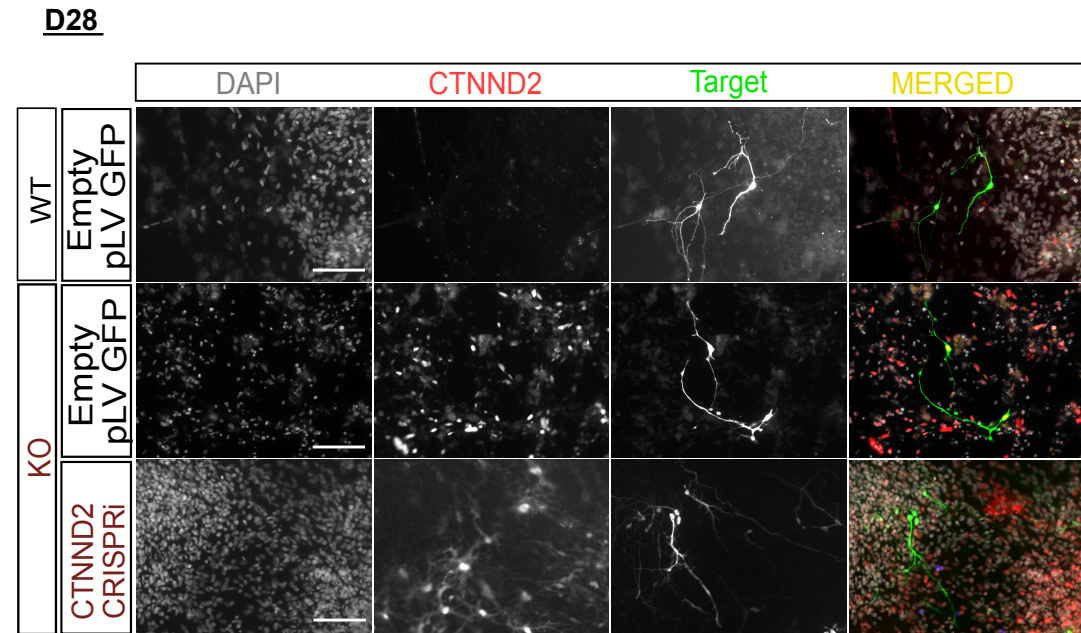

F

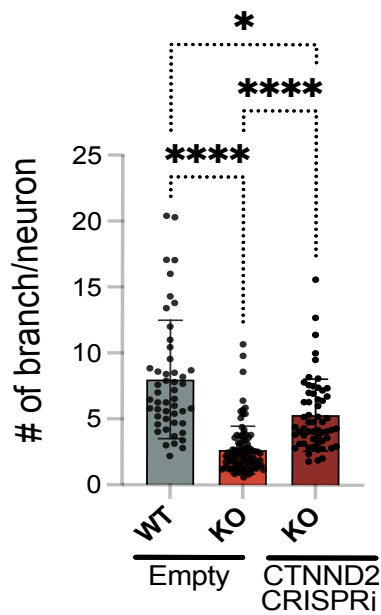

Supplement: S8 Fig — (A) iNeuron formation from iPSCs. (B) Expression of CTNND2 is reduced upon CRISPRi transfection in 293T cells by RT-qPCR. (C, D) Successful neuronal differentiation at different time points (D5 and D14, respectively). Panel (C) shows ICC images of cells that were stained for early neuronal marker Tuj1 (TUBB3) at day 5, whereas panel (D) shows ICC images of cells that were stained for mature neuronal marker, MAP2, and cortical neuronal marker, CUX1 at day 14. (E) ICC images of GFP+ cells that are stained for CTNND2 and CUX1 at day 28. GFP+ cells in the WT and KO condition express empty GFP, whereas GFP+ cells in CTNND2 rescue conditions express dCas9 and gRNA targeting CTNND2. (F) Quantification of neuronal morphology in WT, KO, and CTNND2 CRISPRi conditions. n = 4–5 per sample on glass coverslips were used, and n = 10–14 per coverslip were quantified. Data are represented in bar graphs as mean ± STD with individual data as dots; *p <0.05, and ****p <0.0001 Kruskal–Wallis ANOVA test with Dunn’s multiple comparisons test as a post hoc was used. Scale bar = 100 μM for S8C–S8E Fig. The numerical values that were used to generate the graphs can be found in S1 Data. CRISPRi, CRISPR inhibition; ICC, immunocytochemistry; iNeuron, induced neuron; iPSC, induced pluripotent stem cell; KO, knockout; RT-qPCR, quantitative real-time PCR; WT, wild type. (PDF) [file pbio.3001852.s008.pdf]

# Figure S9

## IPSC WT

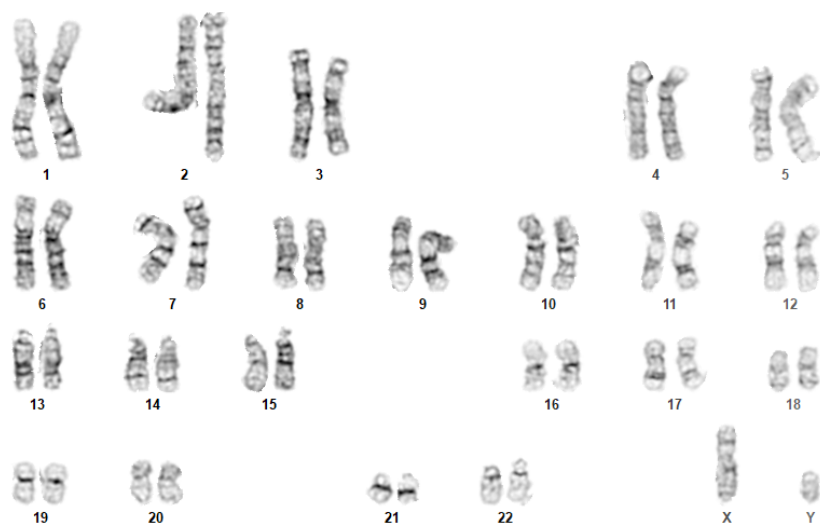

## IPSC KO-1

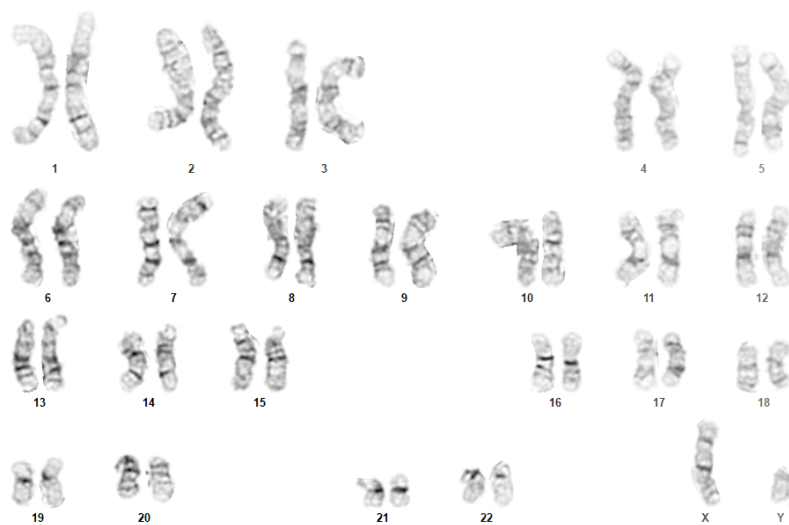

## IPSC KO-2

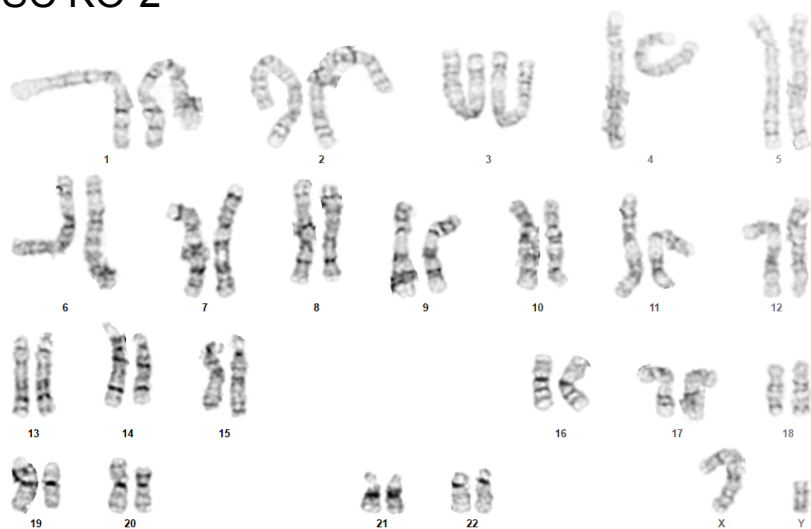

Supplement: S9 Fig — (PDF) [file pbio.3001852.s009.pdf]
