# Supplementary material for: FOXP1 orchestrates neurogenesis in human cortical basal radial glial cells
Source: PLoS Biol. 2023 Aug 4;21(8):e3001852. doi: 10.1371/journal.pbio.3001852 (PMC10431666; doi:10.1371/journal.pbio.3001852)
Supplement: S1 Table — (DOCX) [file pbio.3001852.s010.docx]

| Sample name | Genotype | Total Reads (NextSeq) | Total Reads (NovaSeq) | Total Nuclei | Mean Reads per Nucleus | Median Genes per | Median UMI per |
| --- | --- | --- | --- | --- | --- | --- | --- |
|  |  |  |  |  |  | Nucleus | Nucleus |
| WT_1 | WT | 54,992,799 | 308,137,509 | 13,853 | 26,213 | 1,986 | 3,046 |
| WT_2 |  | 55,999,689 | 329,936,982 | 31,477 | 12,260 | 1,420 | 2,002 |
| WT_3 |  | 59,056,503 | 332,920,320 | 17,569 | 22,310 | 1,902 | 2,846 |
| KO-1_1 | P1 gene body deletion | - | 391,215,611 | 13,066 | 29,941 | 1,870 | 2,837 |
| KO-1_2 |  | - | 376,442,968 | 4,725 | 79,670 | 1,746 | 2,705 |
| KO-1_3 |  | - | 414,955,518 | 12,180 | 34,068 | 1,875 | 2,880 |
| KO-2_1 | FOXP1 KO  Puro KI | 52,016,272 | 300,208,358 | 14,096 | 24,987 | 1,830 | 2,835 |
| KO-2_2 |  | 56,731,346 | 358,688,357 | 30,304 | 13,708 | 1,528 | 2,208 |
| KO-2_3 |  | 60,792,883 | 356,049,243 | 14,066 | 29,634 | 2,251 | 3,805 |
| TOTAL | | 3,508,144,358 | | 151,336 |  | | |

**Table S1. Sequencing read numbers, number of nuclei sequenced, number of expressed genes, and number of UMIs.**
