# Supplementary material for: FOXP1 orchestrates neurogenesis in human cortical basal radial glial cells
Source: PLoS Biol. 2023 Aug 4;21(8):e3001852. doi: 10.1371/journal.pbio.3001852 (PMC10431666; doi:10.1371/journal.pbio.3001852)

Filename: FOXP1_WB_1

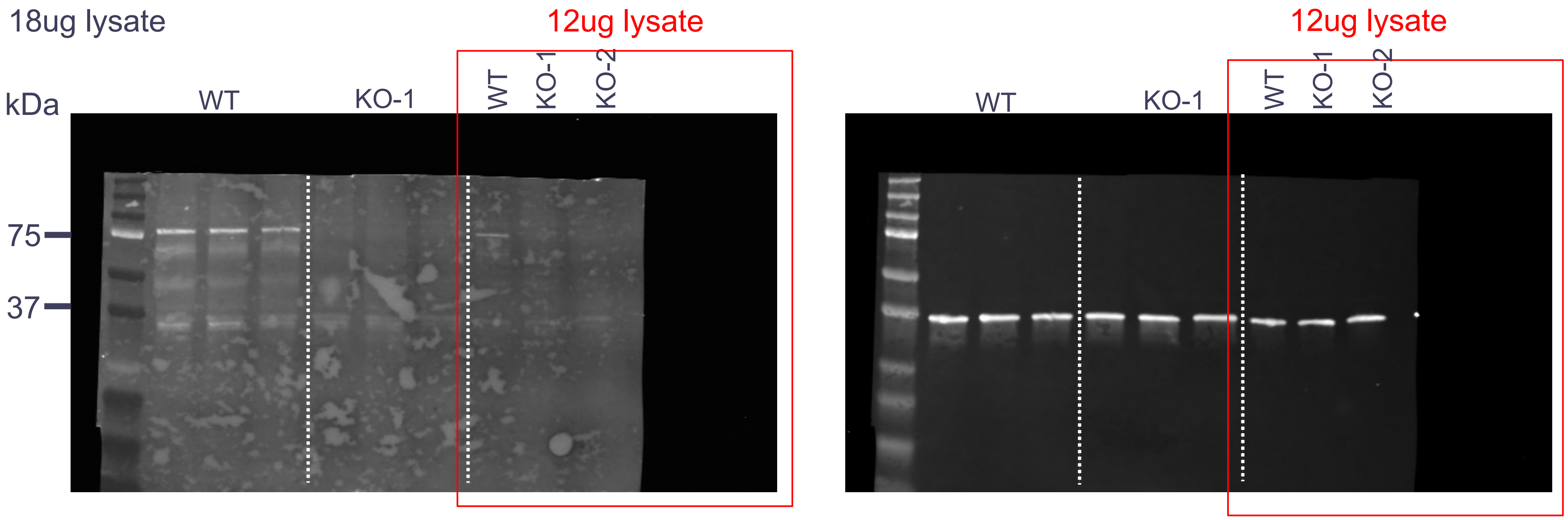

Filename: FOXP1_WB_2

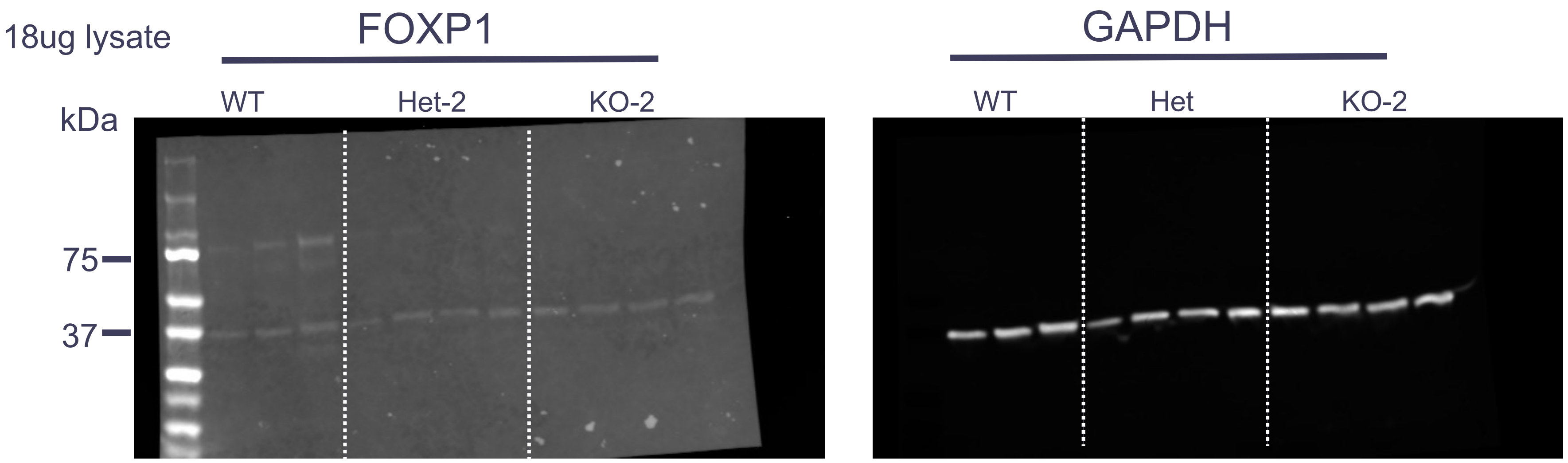

Biorad Dual Color Protein Ladder

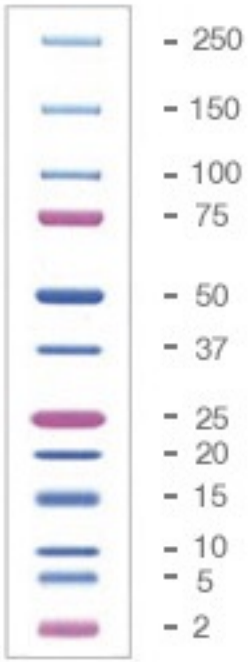

Biorad All Blue Protein Ladder

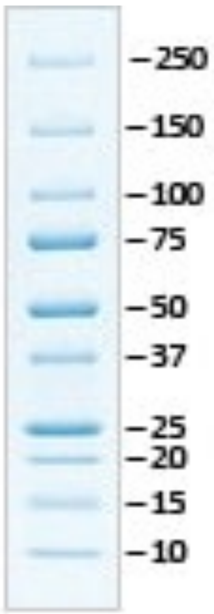

Supplement: S1 Raw Image — (PDF) [file pbio.3001852.s016.pdf]
