# Supplementary material for: FOXP1 orchestrates neurogenesis in human cortical basal radial glial cells
Source: PLoS Biol. 2023 Aug 4;21(8):e3001852. doi: 10.1371/journal.pbio.3001852 (PMC10431666; doi:10.1371/journal.pbio.3001852)
Supplement: Attachment — Submitted filename: Park_Reviewer_Response.pdf [file pbio.3001852.s017.pdf]

We are pleased by the overall interest and enthusiasm of the reviewers for our study. We appreciate the opportunity to respond to the many insightful comments that the reviewers had about our manuscript. Both reviewers have provided constructive feedback to our previous submission. We have addressed each comment in a point-by-point response below. By adding both new analyses and refinement of our previous results, we believe this has resulted in a significantly improved manuscript. Specifically, we have added additional information about aRGCs and our rationale for focusing on bRGCs, new data on CRISPRi rescue experiments in FOXP1 KO cells, more information about proportion of cell types, new data that include high resolution and quantification of FOXP1 in each cell type, and new information/display of genes that denote bRGCs.

Reviewer #1: FOXP1 orchestrates neurogenesis in human cortical basal radial glial cells

Park et al. present a study aimed at understanding the human-specific role of FOXP1 in cortical development and linking its loss of function to human neurodevelopmental disorders. The study is done in cortical organoids, which is advantageous in many aspects to rodent models used in past studies of this gene. The study relies on snRNA data images to depict the gene's role in the developmental trajectory of cortical progenitors, focusing on a subclass of progenitors that occupy the main proliferative zone in the human cortex. The study is of great interest, but it suffers from some problems, particularly the nomenclature of the human progenitor subclasses and the experiments that reveal decreased proliferation and differentiation of bRGCs following FOXP1 loss.

We appreciate the reviewer's positive assessment of the importance of our study. We apologize for any nomenclature issues within our manuscript and have taken steps to correct these throughout.

Specific remarks:

NDD- Neurodevelopmental disorders, the acronym is used without its meaning.

We apologize for not providing the description of the acronym. The reviewer is correct that we used NDD as a term for neurodevelopmental disorders. We have now clearly defined this in the revised manuscript.

Figure 1: Based on the strategy to produce KO2, one would expect to detect GFP expression in the sections. Is it there? (looking at Figure 3B, no GFP is expressed in KO2?). If GFP is expressed in KO2, wouldn't it be a great tool to look at the location proliferation and progeny of the mutated cells or a way to isolate the mutant cells? Why is this option not being used throughout the study?

The reviewer is absolutely correct about the design of KO2, and it was our original intention in our design to use GFP expression to identify the mutant cells. We had some success in identifying such cells with GFP in the snRNA-seq data. However, since we did not make a separate, second library that amplified the GFP signal (as is now becoming the norm in snRNA-seq studies using a transduced tag), the number of nuclei we detected that were tagged in this manner was low. With respect to using any GFP protein signal for IHC analyses etc., unfortunately, while the GFP protein is much easier to observe at the iPSC stage (and we used it to sort for clones), upon differentiation into organoids the expression is only as good as FOXP1 endogenous expression in any given cell type, which itself is not that high (likely due to its function as a transcription factor). In addition, the KO2 GFP construct uses TagGFP2, which does not amplify with a normal GFP antibody. Thus, this is not a confound for the experiments in Figure 3 with Ad-eGFP, in which you can see GFP without amplification. We now add this caveat and improve details in our Methods section as below:

"The second FOXP1 KO (KO-2) cell lines were generated using the CRISPR Paint strategy (Schmid-Burgk et al., 2016). We used 'pCRISPaint-TagGFP2-PuroR' plasmid (#80970) to insert 'TagGFP2-2A-PuroR-PolyA' into the FOXP1 locus. (Upon differentiation into organoids, the GFP signal is expressed at low levels in the KO-2 cells and requires the use of a TagGFP2-specific antibody.)" Below are our staining results showing expression of TagGFP2 amplified with TagGFP2 antibody.

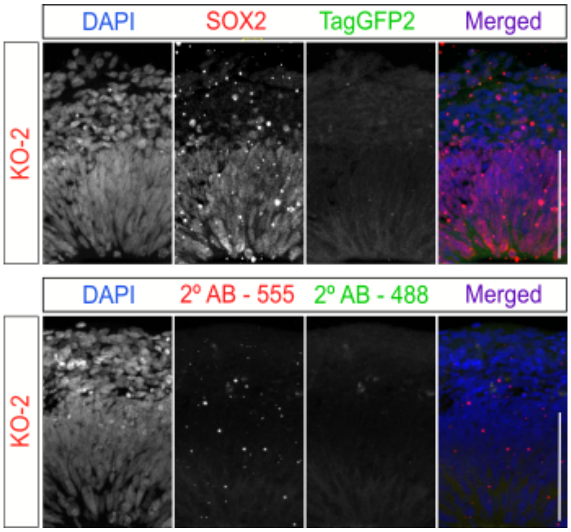

The top image shows the result of ICC staining using DAPI, anti-SOX1, and anti-TagGFP2. There is weak TagGFP2 staining throughout this representative KO-2 organoid. The bottom image was stained with DAPI and the corresponding IgG controls and secondary antibodies for the top images in order to show the background level for SOX2 and TagGFO2.

Figure S1/Figure 2: Based on others, D25 oRG's are rare at the developmental time, yet FOXP1 expression is abundant in the VZ. Likewise, at D40 (Figure 1D), the VZ is the prominent proliferative region, and only a few FoxP1 cells are seen in more basal locations. Previous studies show HOPX and FOXP1 colocalize to cells in the osVZ in human sections. Given this, why do the authors consider FOXP1 to be a bRG-enriched gene in their organoids? With the small proportion of bRG's at D40 when most of the study was conducted and the high expression in cells that are not bRG, I find the statement the study focuses on bRG a little problematic.

We apologize if this was not clear. We did not intend for readers to think that FOXP1 is bRGC-enriched relative to other cell types. We focused on bRGCs because we thought this was an advantage of the human organoid system over model systems such as mouse (as the reviewer points out in the beginning of their review). So, yes, while we could have focused on any number of cell types that express FOXP1 in the organoids, we thought it was most exciting to focus on a cell type that has differential FOXP1 expression in humans and rodents.

At D40, FOXP1 is expressed in all cells in the organoids, but its expression is higher in the progenitors, which is why it appears in Figure S1 to be primarily expressed in progenitors. Based on ICC staining using cell-type specific markers, we observed FOXP1 expression in 95.25% of aRGCs, 87.25% of bRGCs, 71.25% of IPCs, and 52% of ENs (Supp Fig 1E-F). This is comparable to what we observed in our snRNA-seq dataset, which showed 72% of aRGCs, 79.35% of bRGCs, 87.1% of IPCs, and 49.6% of ENs express FOXP1 in WT organoids (Supp Fig 2A and 2C).

To be clear about why we focus on bRGCs, we say:

“To capture when FOXP1 expression is abundant in the basal cortical progenitors that can give rise to upper layer neurons where FOXP1 is also ultimately expressed (Pearson et al., 2020), we performed the majority of our experiments at D40 ± 1-2 days (Mora-Bermúdez et al., 2016; Otani et al., 2016). Other work has reported outer radial glial cells in human brain organoids at around this same time (Rosebrock et al., 2022). An advantage of using human brain organoids to examine FOXP1 function is the potential presence of bRGCs, which are not as abundant in rodents and do not express FOXP1 (Pearson et al., 2020).”

Figure 2. In the beautiful layered images of D40 (Figure S1C) it seems like the developmental trajectory is FoxP1+(aRG?)-> TBR2+(IPC)->CTIP2+(EN). The scRNA data shows a pseudo time in with aRG->bRG->IPC->EN. Is FOXP1 better defined, at this developmental stage, as a typical aRG rather than a bRG gene?

The confusion is partly due to the nomenclature used (more suitable for rodent brains rather than human brains). Based on some papers that the authors cite (Kanton et al., 2019; Nowakowski et al., 2017), the early cortical development trajectory is RG->IPC->EN, and in later stages, RG-> oRG->IPC (both oRG and IPC have the neurogenic ability). Later, RG will give rise to tRG, which will contribute to a noncontinuous scaffold with the basal processes of the oRG cells. In this study, the authors state that they aim at the beginning of neurogenesis. Yet, the cell line they are focusing on is highly neurogenic at later stages, and the trajectory depicted is aRG->bRG->IPC, consistent with late neurogenesis.

We understand the reviewer's concern about the developmental timing. Because of the sensitivity of snRNA-seq to do a genome-wide assessment, we are able to identify distinct aRG and bRG clusters.
As we are sure the reviewer knows, ENs can be derived from aRGCs, bRGCs and IPCs (Breunig et al., 2011, Lui et al., 2011).). This has been detected in the developing human cortex as early as the early second trimester (Eze et al., 2021; Pollen et al., 2015). Below is a published scRNA-seq dataset from human fetal cortex (Nowakowski et al., 2017), in which oRGCs appear as early as gestation week (GW) 12-13. This is approximately the time when expression of FOXP1 is observed in the progenitors primarily as well as in some intermediate progenitors (Pearson et al., 2020).

Nowakowski et al., 2017

oRG cells (=bRG cells) **Cell types at PCW 14-15 (= GW12-13)** **FOXP1+ cells**

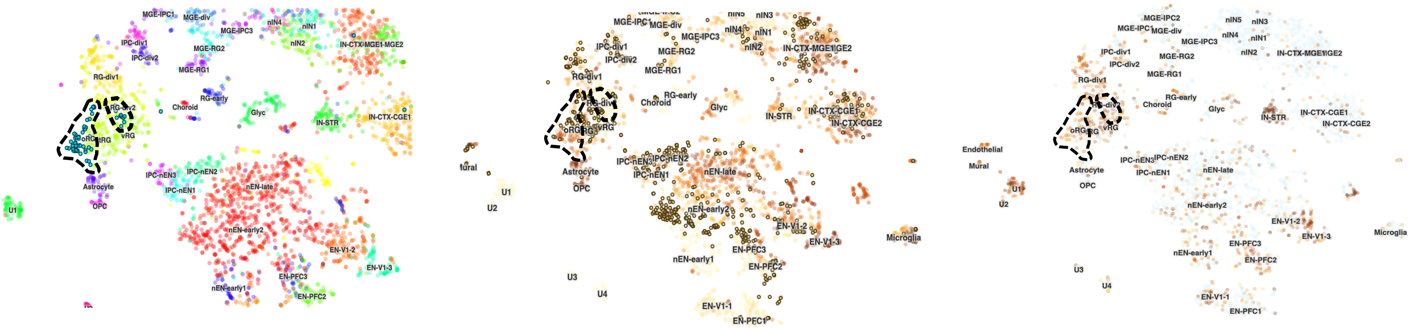

Unlike rodents, this mechanism is known to be more complex in humans (Figure below from Florio and Huttner 2014), which is why we wanted to use the human organoid system. Studies have suggested that early neurogenesis gives rise to neurons in the deep and upper layer cortical neurons whereas later neurogenesis gives rise to neurons in the upper layer cortical neurons (Franco et al., 2014, Shi et al., 2021). We wanted to determine how the absence of FOXP1 affects early neurogenesis starting in the multipotent progenitor cells. However, we note that FOXP1 is primarily expressed in upper layer neurons, so our focus on including the bRGC distinction also makes sense for a study investigating FOXP1 function.

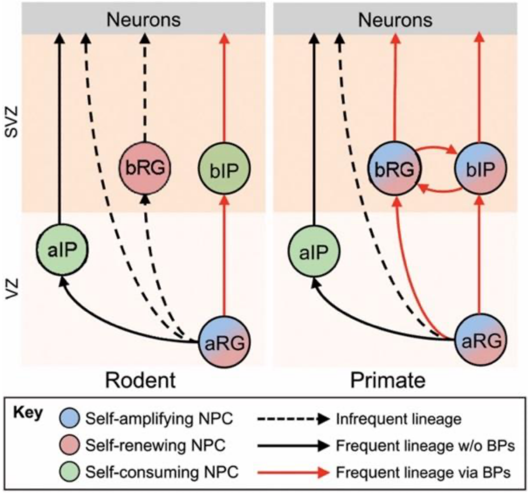

From Florio and Huttner 2014.

tRGCs are indeed an interesting cell type to us as well. However, while tRGCs are thought to be important for prolonged neurogenesis in the primate cortex (Pollen et al., 2015; Nowakowski et al., 2016), there is currently no evidence suggesting that tRGCs are specifically linked to human cortical expansion. In our snRNA-seq dataset, we detected a cluster that was annotated as tRGCs when we integrated our dataset with a published human fetal cortex dataset and another published brain organoid dataset. The tRGC cluster was only observed in this integration analysis and not when using other analysis methods. Additionally, although a large percentage of these cells expressed *FOXP1*, the number of cells constituting the tRGC cluster was relatively small. Based on the uncertainty of this result, we decided not to describe tRGCs in our dataset.

Figure S2: “We observed FOXP1 expression in 79.35% of the bRGCs (Figure S2C)”. In figure S2, which are the bRGs? The figure shows RG, IPC, and EN. Can a list of genes defining clusters 17 and 25 (Figure 2) be presented in the main figure?

We apologize that this is indeed not clear from Figure S2. We have now replotted these data with new labels. These new plots now show more clearly the contribution of either bRGCs or aRGCs in Figure S2A, C, and D.

We also apologize if the description of clusters 17 and 25 as bRGC-like was not clear. We had stated:

“We identified two bRGC clusters (clusters 17 and 25) (Figure 2A-B) based on the annotation (Figure S3C). We further confirmed the cell type identity of these two clusters by performing a Fisher’s exact test against a list of bRGC-enriched genes derived from a previous study (Heng et al., 2017) (Figure 2C).”

Thus, clusters 17 and 25 were defined as bRGC clusters in a number of ways. First, we used the Nowakowski et al., 2017 snRNA-seq data and performed a Fisher’s exact test to identify significantly overlapping clusters with the cluster they defined as “RG_oRG”. These results are in Figure S3C. Second, we again used a Fisher’s exact test with the Heng et al., 2017 data to further confirm the validity of cluster 17 and 25 as bRGC-enriched (Figure 2C) using the list of genes from the Heng et al. meta-analysis of bRGC gene expression.

Therefore, the identification of these two clusters as bRGC-like are not specifically defined by a list of genes. However, we do show violin plots for clusters 17 and 25 in Figure S3D of expression of four genes (*PTPRZ1*, *LIFR*, *TNC*, and *HOPX*) that are frequently expressed in bRGCs.

We also include a list of genes that are highly expressed in these clusters from a meta-analysis of the literature of bRGC-relevant gene expression in new Figure 4F and Tables S4 and S5.

Figure 3:
The study by Pearson et al (https://www.ncbi.nlm.nih.gov/pmc/articles/PMC8397815/) showed that Foxp1 promotes aRG maintenance in the mouse cortex by promoting vertical, symmetric self-renewing cell divisions. I expected the study of FOXP1 on the proliferation and progeny of aRG and bRG to be conducted with extra care. Indeed, the strategy to use viruses for sparse labeling is very suitable for the study. Yet, the identification of bRG is based on morphology and negative criteria of low expression of Tbr2. The morphology study is done on 16 □M thick slices, which may be why many processes are not continuous (Figure 3B). Given the 3D nature of the organoid VZ, I believe that a thick slice will be more reliable.

We followed a method published in the literature (Andrew et al., 2020). Moreover, we compared slice thicknesses of 10uM, 20uM and 30uM. 10uM was good for nuclear staining such as transcription factors but too thin for examining cellular processes, whereas 30uM was too thick for examining single nuclear staining clearly. 20uM was best for co-staining with nuclear markers as well as for examining cellular processes. We processed WT and KO slices together on the same day on the same slides from growing cells to fixation, sectioning and staining. Lastly, upon comparing EGFP+ cells using slice thicknesses between 20uM and 30uM, we found that thickness does not matter as much as the sparsity of the virus.

Furthermore, morphology is problematic in later stages; the authors show that in the FOXP1 KO, adhesion molecules are down-regulated. Would that potentially cause changes in possesses morphology?

The reviewer brings up a very interesting point that we had not previously considered. Adhesion molecules have been a fascinating interest to us as well as FOXP1 and its partner FOXP2, are known to regulate adhesion molecules (Rousso et al., 2012). When we were examining genes differentially regulated in each major cell type – such as the components of the WNT, mTOR, and Notch signaling pathways we saw catenin being one of the gene classes that is most consistently differentially regulated across many EN clusters. Catenins in the WNT signaling pathway serve as an integral structural component of cadherin-based adherens junctions between the neighboring cells as well as the key nuclear effector of the canonical Wnt signaling pathway in the nucleus (Valenta et al., 2012). *CTNND2*, in particular, is a gene that encodes for δ-catenin (Turner et al., 2015). It is also a high-confidence ASD gene in the SFARI database. *CTNND2* is most consistently differentially upregulated in many of the EN clusters in our snRNA-seq dataset. We now add discussion of this as a caveat:

*"CTNND2, a gene encoding for δ-Catenin that is involved in WNT signaling through its interaction with N-cadherin as well as neurite outgrowth and function (Bareiss et al., 2010; Nopparat et al., 2015; Schaffer et al., 2018; Zhang et al., 2018), is upregulated in EN clusters. Since the loss of Foxp1 leads to impaired dendritic and axonal formation (Braccioli et al., 2017; Li et al., 2015), we asked whether elevated levels of CTNND2 would result in a similar phenotype as the loss of FOXP1 in the cortical neurons. Therefore, we performed a rescue experiment on the neuronal morphogenesis-related phenotypes by applying a CRISPRi approach to CTNND2 (**Figure S8A-B**). Using a published protocol (Zhang et al., 2013), we generated 2-D cortical neurons that express TUJ1, MAP2 and CUX1 (**Figure S8C-D**) as well as SATB2 and vGLUT2 (not shown), which are the markers that are expressed in FOXP1+ cortical neurons during the mid-late neurogenesis as well as post-mitotically. FOXP1 KO cells exhibited significantly fewer developed dendritic branches compared to WT. Inhibiting CTNND2 using CRISPRi rescued this phenotype to a significant level (**Figure S8E-F**). We believe this result could provide us with a better understanding of how FOXP1 orchestrates neurogenesis by regulating WNT signaling in the developing brain."*

We now include these data as new Fig. S8.

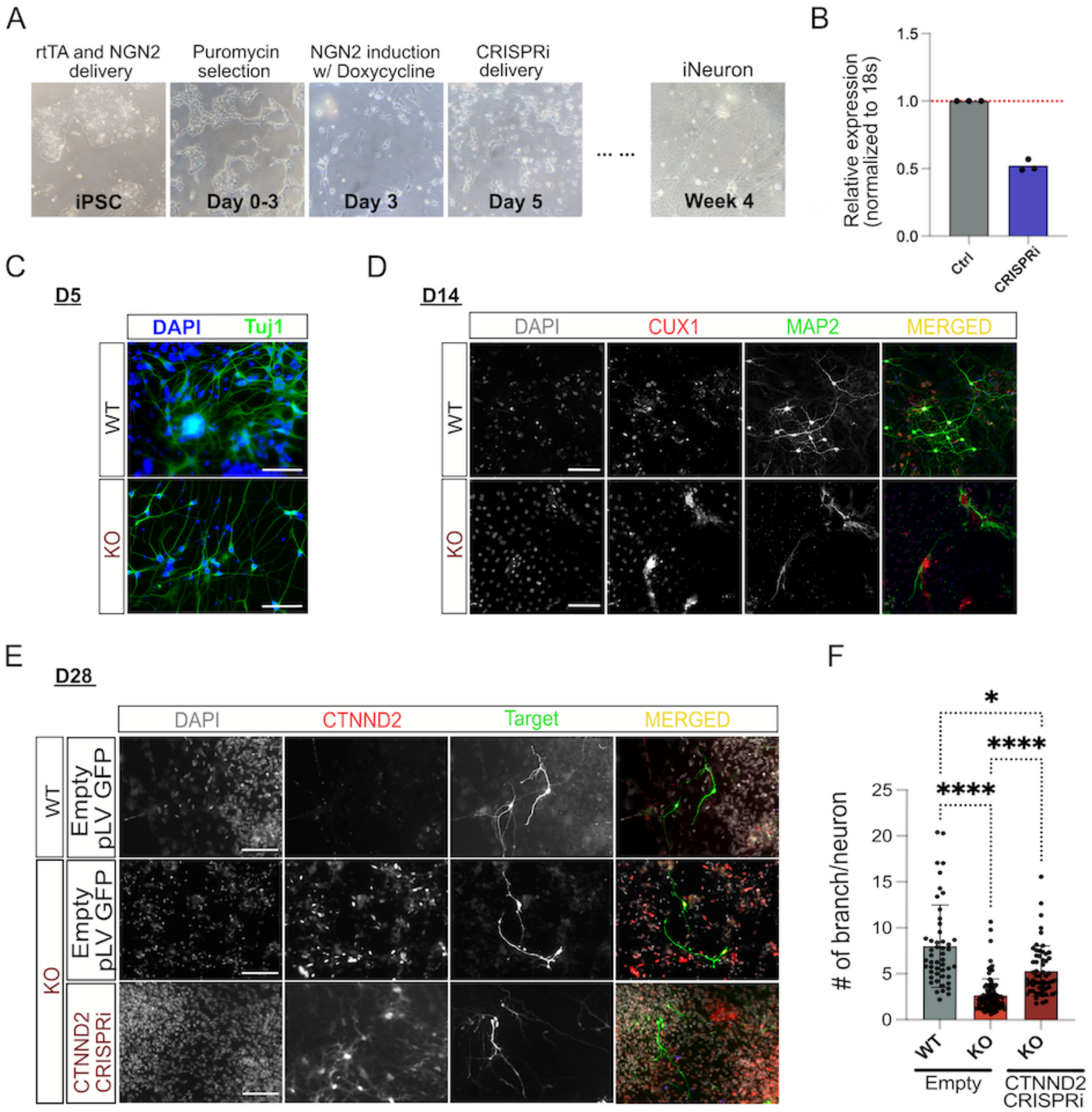

Figure S8. Neuronal morphology rescue by inhibiting expression of CTNND2. (A) Induced neuron (iNeuron) formation from iPSCs. (**B**) Expression of CTNND2 is reduced upon CRISPRi transfection in 293T cells by qPCR. **(C)-(D)** Successful neuronal differentiation at different time points (D5, D14 respectively). Panel (**C**) shows ICC images of cells that were stained for early neuronal marker Tuj1 (TUBB3) at day 5. Panel (D) shows ICC images of cells that were stained for mature neuronal marker, MAP2, and cortical neuronal marker, CUX1 at day 14. **(E)** ICC images of GFP+ cells that are stained for CTNND2 and CUX1 at day 28. GFP+ cells in the WT and KO condition express empty GFP whereas GFP+ cells in CTNND2 rescue conditions express dCas9 and gRNA targeting CTNND2. **(F)** Quantification of neuronal morphology in WT, KO and CTNND2 CRISPRi conditions. Data are represented in bar graphs as mean ± STD with individual data as dots; *p <0.05, and ****p <0.0001 Kruskal-Wallis ANOVA test with Dunn's multiple comparisons test as a post hoc was used. Scale bar = 100uM for Supp Fig 8D and 8E.

We now include the following in the Methods:

Neuronal morphology rescue experiment using CRISPRi strategy

WT and KO iPSC lines were differentiated into 2-D induced neurons based on published protocols with some optimizations necessary for our own iPSC lines (Zhang et al., 2013, Frega et al., 2017). The modifications are as follow: we coated the plates with Poly-O-Laminin the night before and then coated the plates with Matrigel for 1 hour at 37 degree Celsius prior to plating the cells on glass cover slips. We fed the cells Laminin a concentration of 5ug/ml every 2-3 days. The concentration of Puromycin varied depending on the stage of differentiation and we typically used somewhere between .3-1ug/ml. Reverse tetracycline-controlled transactivator (rtTA) (FUdeltaG@-rtTA; addgene, #19780) and NGN2 (pTet-O-Ngn2-Puro; Addgene, #52047) were purchased from Addgene. Lentivirus infected cells were selected for expression of the puromycin resistant gene. Cells were plated, differentiated and maintained on glass-cover slips in 24-well plates for up to 28 days. Rescue experiments using CRIPR inhibition (CRISPRi), which was designed to inhibit the expression of CTNND2 using dCas9, began on day 6 when the cells were transitioning from progenitor medium to differentiation medium and were examined on day 28. Targeting cells with CRISPRi was done sparsely with a GFP reporter downstream of gRNA to examine the morphology of neurons thoroughly. sgRNA pairs were designed using CRISPick (Concordet and Haeussier, 2018; Labun et al., 2019). F: 5'-ACCGGTCCAGGGCGTGCGTTCCCA-3' and R: '5- AACTGGGAACGCACGCCCTGGACC-3' were designed to be 131 bp away from the known TSS of CTNND2. The sgRNA sequence with the top four fewest off-target sites in the human genome was selected for use. The pair of sgRNA was cloned into pAAV-U6-CMV-GFP (Addgene, #R0569). All the virus packaging work was performed by Neuroconnectivity Core at Baylor College of Medicine Intellectual and Developmental Disabilities Research Center. To ensure successful neuronal differentiation, we checked for PAX6, Tuj1, MAP2, SATB2, vGLUT2, and CUX1 expression on days 5, 14, and 28. All the WT and KO cells were grown under the same conditions, and dendritic complexity analysis was performed following a published method (Rosario et al., 2012) across both WT and KO cells.

The second part of the figure is even more problematic. Staining with HOPX reveals the low abundance of bRGs at the time point but also raises the question of why this was not used as a positive criterion in Figure 3A to identify the bRG that are supposed to be the center of the study. The progeny study is based on location and is not reliable, in my opinion. Given that a viral infection is so elegantly done, clonal analysis of the sparsely infected cell is more suitable.

We agree with the reviewer that using a reliable gene marker as a positive criterion would have been preferable than using a negative criterion such as reduced TBR2 expression and location as well as morphology (Bershteyn et al., 2017; Sauerland et al., 2018; Schutze et al., 2023) as we did. And we indeed include data on HOPX expression in Figure S5. The issue is that while HOPX is expressed in bRGC but not IPCs or aRGCs, HOPX is not exclusively expressed in bRGCs (Andrews et al., 2020 and Matsumoto et al., 2020). In fact, to our knowledge, the field has not determined a single gene that could be used to mark all bRGs exclusively in human tissue. This is why we used genome-wide enrichment to determine the clusters of nuclei that are likely bRGs in the organoids. Unfortunately, we cannot apply such an approach to our immunostaining and thus had to make a challenging decision on how to identify these cells conservatively. We believe this approach is indeed in line with what Pearson et al did in their study.

Reviewer #2: Park et al., present their work investigating the role of FOXP1 in basal radial glial cells during human corticogenesis, using cortical organoids derived from FOXP1-/- iPSCs. This work is novel and significant as it begins to define the role of FOXP1 in human cortical progenitors, and performs scRNA Seq to assess the impact of loss of FOXP1 in human cortical cell populations. A particularly interesting observation is that there are 2 identified populations of FOXP1+ bRGCs and that FOXP1 may regulate different pathways in each population. Also, that bRGC may be especially vulnerable to ASD. However, I have issues with the authors interpretation of their results that need to be addressed.

We thank the reviewer for the very positive assessment of our work and its potential impact on the field. We have now substantially edited the manuscript to address concerns about data interpretation.

The statistical analyses and supplemental data are sufficient as are the details in the materials and methods.

We thank the reviewer for this positive feedback on the statistics, methods, and supplemental data.

Minor concerns/edits:

* Authors mix up descriptions of direct and indirect neurogenesis in the 1st paragraph of the introduction.

We apologize for this typo and have corrected the sentence:
"bRGCs are known to produce neurons through both indirect neurogenesis, a production of neurons via intermediate progenitors, and direct neurogenesis, a production of neurons directly from bRGCs."

* The experiments are performed using day 40 organoids, however there appear to be very few bRGCs present at this time point – at least as shown by the immunohistochemical analyses in Fig 1 and S1. The image quality in general needs to be improved as it is difficult to see some of the stains at the current resolution.

We apologize for the image quality and have now uploaded individual high-resolution images. At day 40, based on our snRNA-seq data in WT organoids there are 3.8% of total cells that we define as bRGCs. Below is a summary of % cell type breakdown as well as the expression level of FOXP1 in each cell type based on the pseudobulk analysis of our snRNA-seq dataset. Please keep in mind that while the overall percentage of bRGCs is relatively low, this represents almost 5,000 nuclei in our dataset. In addition, roughly 75% of the bRGCs (in WT) are positive for FOXP1.

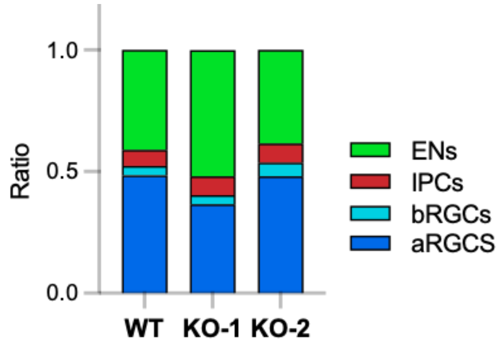

CELLTYPE	WT	KO-1	KO-2
aRGCs	48.5%	48.1%	36.6%
bRGCs	3.8%	5.6%	3.8%
IPCs	6.7%	7.9%	7.6%
ENs	41.1%	38.4%	52.0%

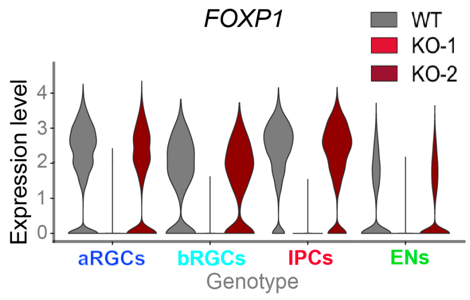

* The authors should include improved images of FOXP1+ bRGCs in their organoids.

We now provide new high-resolution images that show expression of FOXP1 in bRGCs and quantification of these cells as defined by FOXP1+TBR2- and bRGC morphology based on EGFP expression (Supp Fig 1E and F).

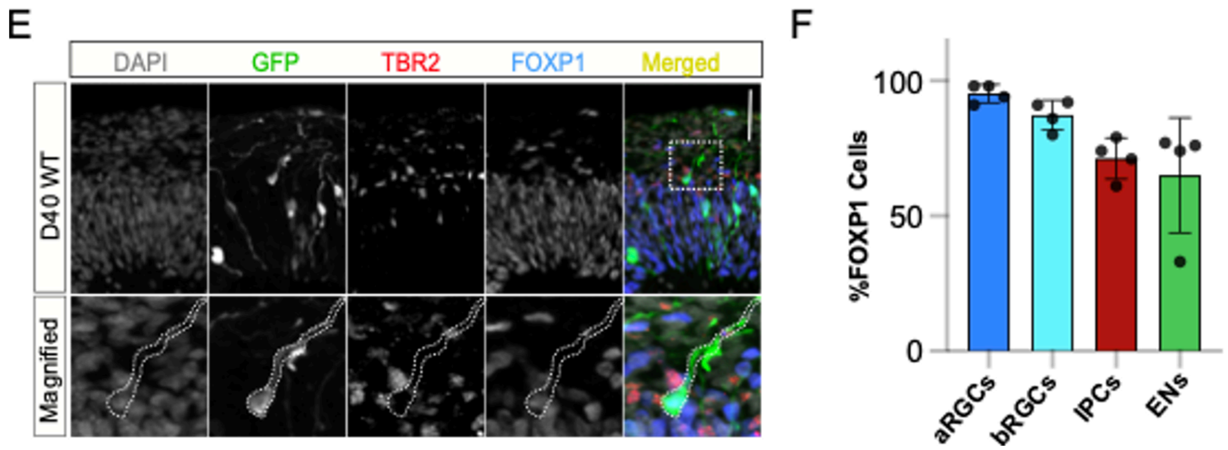

(E) Detection of 'ad-EGFP' infected FOXP1+ bRGCs based on morphology, location and absence of TBR2 expression, **(F)** Quantification of FOXP1+ aRGCs, bRGCs, IPCs and ENs**.** Scale bar = 50uM for panel E. n=4 cortical structures used from WT organoids.

* Figure 2 – the authors use expression of SOX2+PAX6+HES5+ transcripts as 0 in their pseudotemporal analysis to represent aRGCs. However, these trancripts should also be present in bRGCs. I found the descriptions of Figure 2 difficult to follow.

We apologize if the description of Figure 2 is hard to follow. We did indeed set the root of the pseudotime to cells that co-express *SOX2/PAX6/HES5*. Indeed, the root cells are not the only cells that express those three markers as you can see in the feature plots we provide below. However, the root cells express the greatest levels of *SOX2+PAX6+HES5+* but lack any other cell-type specific markers such as *HOPX* or *TBR2* (*EOMES*) as shown in the below feature plots. The set of genes we ultimately assigned as the root cells were derived from Di Bella et al., 2021.

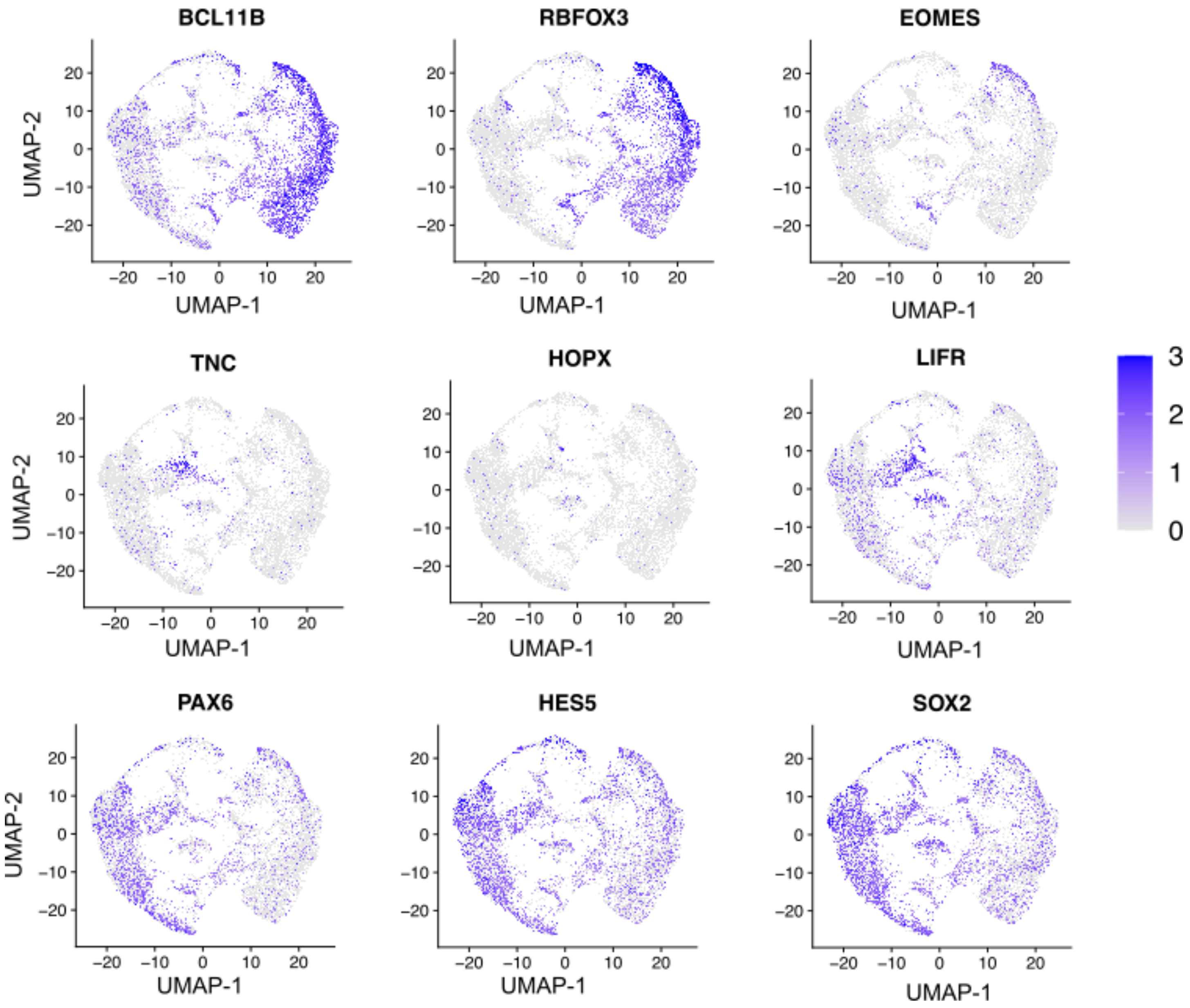

* Figure 2E the authors say “the bRGCs showed reduced gene expression changes from root cells with the loss of FOXP1’ however the density plots suggest a shift towards 0 for bRGCs.

A shift to 0 means the expression of genes did not change much from 0 but this does not necessarily mean that proliferation specific genes are unchanged. In order to assess this, we included a list of our DEGs and our interpretation of the DEGs in the discussion section as well as in the main text in regards to Figure 4 and Table S2 and S3.

* The authors conclude that Figure 2 suggests “impaired differentiation” of bRGCs with loss of FOXP1. This is vague – how is it impairing differentiation? The authors report that certain bRGC genes are decreased – does this suggest the bRGC have differentiated precociously or are more “stem-like”?

Thank you for pointing this out. This is a great series of questions that we also explored. Based on the reviewer comment, we updated this sentence to say:

“Together, these results suggest either impaired differentiation or promotion of a more stem-like phenotype of bRGCs with loss of FOXP1. The bRGCs in the organoids with loss of FOXP1 may precociously

*differentiate into IPCs and contribute to the increased number of IPCs (**Figure S6C**) and to depletion of early progenitors. This, may in turn, lead to decreased numbers of ENs later on in development as reflected in our D100 organoids (**Figure S7**).”*

Indeed “impaired” can be a vague term however, in this case we decided to use it because loss of FOXP1 seems to lead to different changes depending on the cell type. We pointed this out in the manuscript. Moreover, impaired differentiation into neurons does not necessarily mean that the cells are stuck in the proliferation stage. Pseudotime analysis of our scRNA-seq data seems to suggest that there are overall fewer changes from the root aRGCs. However, that does not mean that the self-renewal-pathways in bRGCs remain similar to aRGCs. This led to our quest to understand what individual genes and pathways related to proliferation, differentiation, morphology and neural functions are indeed affected with loss of FOXP1 by examining DEGs in each cell subtype based on snRNA-seq. We provide a list of DEGs in Figure 4 and Tables S2, S3 and S4. We provided our interpretation in the main text as well as in the discussion. Additionally, in order to explore the phenotype resulting from these gene expression changes, we examined the self-renewal and differentiation capacity of bRGCs (Figure 3D-H). Our overall conclusion is that the number and self-renewal capacity of bRGCs and bRGC differentiation are decreased.

* Figure 3 – using low Tbr2 as a marker of bRGCs is subjective. Why not use Pax6 or Hopx?

We used positive expression of HOPX, PTPRZ1, PAX6, and negative expression of TBR2 as well as the typical bRGC morphology to identify bRGCs in this manuscript. We followed methods and knowledge of identifying bRGCs described in the published literature (Bershteyn et al., 2017; Sauerland et al., 2018; Schutze et al., 2023). We also now say:

*“While our criteria for selecting bRGCs may not capture all different types of bRGCs reported in the literature (Andrews et al., 2022), we had at least observed bRGCs with two distinct gene expression profiles based on snRNA-seq **(Figure 2)**.”*

* At the magnification used in some of these images it is difficult to distinguish whether the bRGCs are outside of the ventricular zone. Especially as the images are fuzzy.
* The images in 2E are difficult to interpret due to low quality. The images in 2G should be in color to show merged image. It is not clear that the HOPX+ cells analyzed are outside of the ventricular zone (where bRGCs should reside).

Based on previous imaging studies done (Noctor et al., 2004), it is our understanding that progenitors are not in a fixed location when they divide or are actively migrating. Therefore, we included cells that do not directly touch the basal surface but fit the characteristics of bRGCs otherwise.

We believe the reviewer means Figure 3E and 3G. We now include high resolution images and a color merged image.

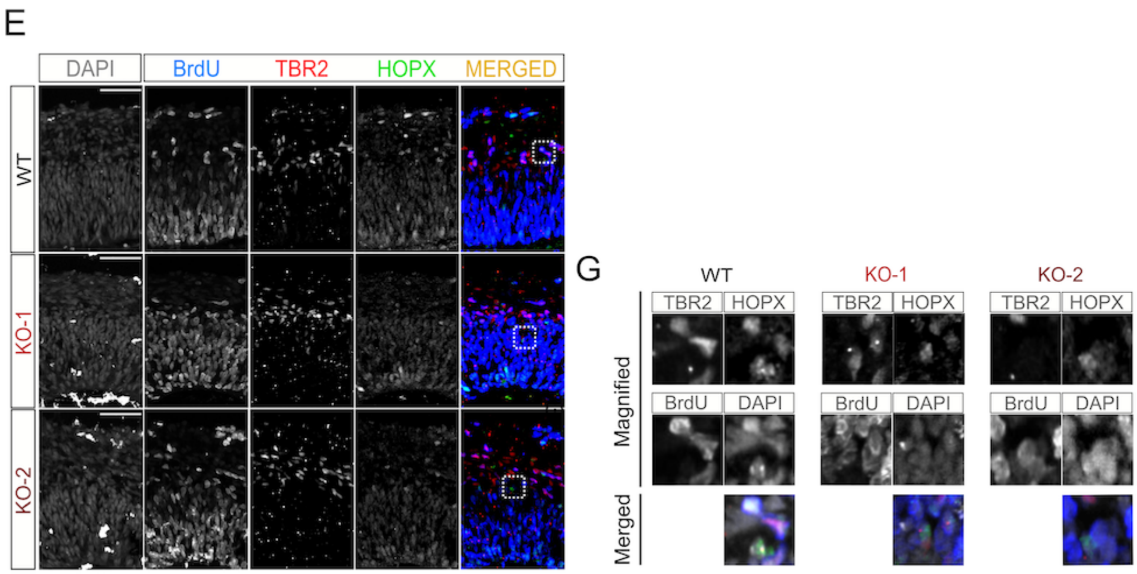

* Figure S5 – the PTPRZ staining does not look specific to bRGCs.

We agree that the PTPRZ staining is not ideal. Also, PTPRZ is not supposed to be specific to bRGCs which is why we used criteria that selected an overlap of PTPRZ/PAX6 while negative for TBR2.

* Figure 3 – the authors should quantify the % of BrdU+ HOPX+ cells over HOPX+ cells rather than DAPI+ to give a more accurate reflection of the proliferating bRGCs. Also, the authors appear to imply that a BrdU+ bRGC is self-renewing, however this assay cannot be used to determine self-renewing capacity, only that the cell is proliferative.

Thank you for pointing this out to us. We now include new quantification per the reviewer's request (Fig 3H). We have also removed reference to self-renewing with respect to our results.

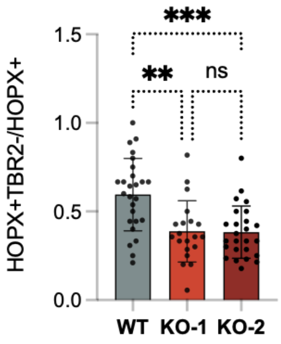

* Figure 3 would be improved by an analysis of the total numbers of aRGCs, bRGCs, Ins and neurons in wt and FOXP1-/- organoids.

We thank the reviewer for this comment, and we have now calculated the total number of each cell type and report these values in the Supplementary Figure 2C, F.

* A more thorough analysis of the other neuronal populations is required to make a statement regarding impact of FOXP1 loss on neurogenesis.

We apologize but we do not understand what other neuronal populations the reviewer is referring to. However, we have quantification of subclusters that express distinct transcriptional profiles (Fig S2A, C, and D). Based on the literature, we believe our D40 organoid embodies represents the developmental stage when neurons with fate as both lower or upper layer neurons is occurring. (Desai et al., 2000; Kaplan et al., 2017; Eze et al., 2021; Longo et al., 2021; Close et al., 2021).

* It seems a shame that the data in Figure S8 C is not in a main figure.

We have now moved Figure S8C to Figure 4 as requested by the reviewer; please see below.

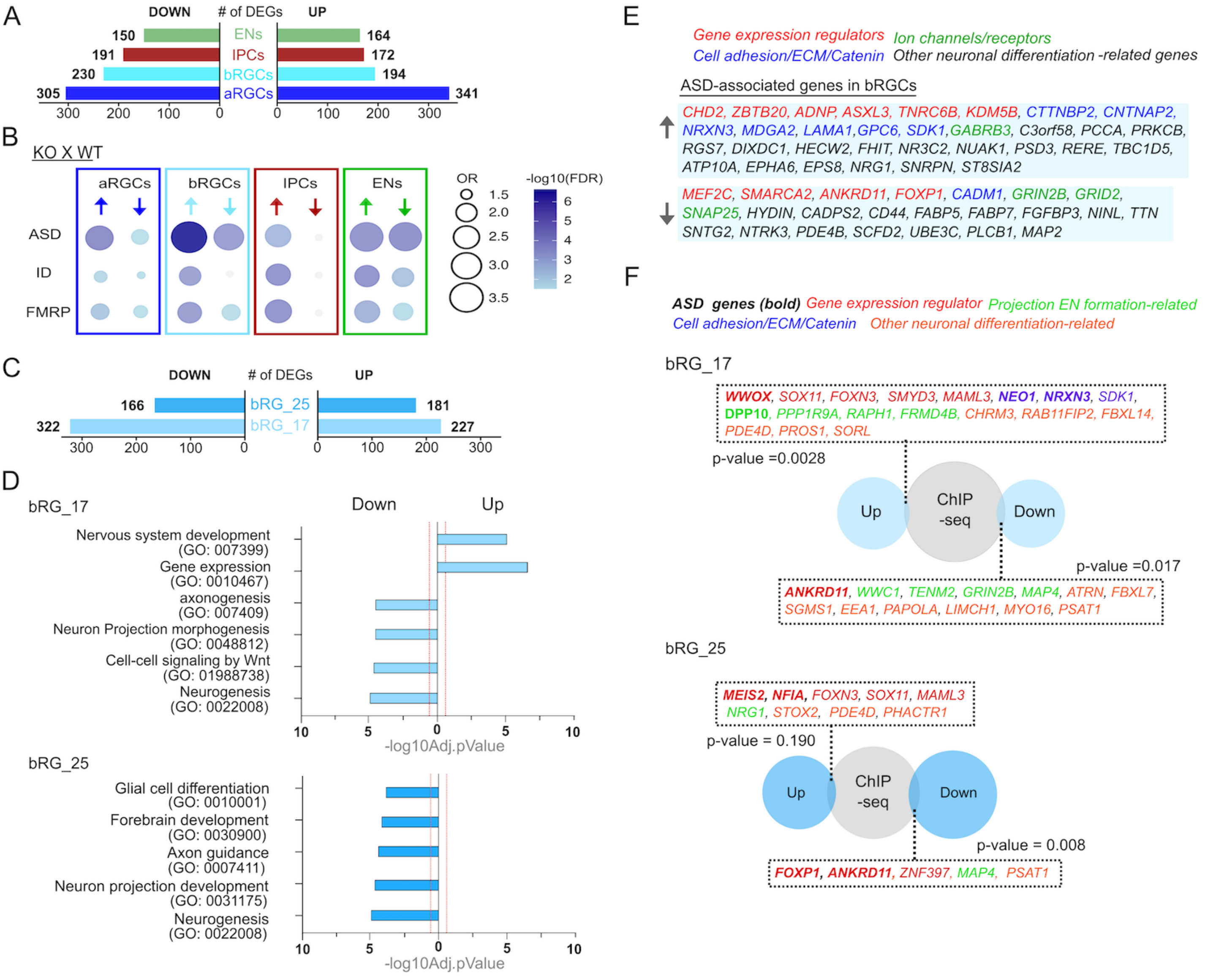

Major issues:

* The analysis in Figure 3 of adjacent cells relative to HOPX+ cells cannot be used to determine indirect or direct neurogenic divisions. To infer whether bRGCs have undergone a direct or indirect neurogenic division, clonal analyses need to be conducted using time lapse imaging with sparsely labelled GFP+ positive cells. The proximity of one cell to another does not indicate that one cell gave rise to the other.

We agree with the reviewer that time lapse imaging would be more definitive than our approach. As we do not have the required live imaging equipment, we instead followed the methods published in the literature that carry

out similar analyses without live imaging (Martínez-Martínez et al., 2016; Li et al., 2020). We now cite these papers appropriately in the revised manuscript. We also soften our interpretation of these data by saying:

*“Therefore, we counted TBR2+ cells located asymmetrically to HOPX+ cells, which represented neurogenic IPCs that we presume underwent indirect neurogenesis based on similar approaches in the field (Martinez-Martinez et al., 2016), and we observed fewer of these cells in the KO **(Figure 3I)**. When we examined HOPX+ bRGCs located asymmetrically to CTIP2+ENs, which represent direct neurogenesis from bRGCs to ENs, we did not find significant differences between the genotypes **(Figure S5C–E)**. Together, our data show that loss of FOXP1 may reduce the proliferative capacity of bRGCs, and also negatively affect indirect neurogenesis from bRGCs to IPCs, but not direct neurogenesis from bRGC to ENs. We did not observe changes in the number of neurons or the number of EN cells that exited the cell cycle to become neurons at this stage **(Figure S6C, F and G)**.”*

* The major issue I have is the failure to consider that FOXP1+ bRGCs are derived from FOXP1+ aRGCs which will also be impacted by the mutation. The authors cannot distinguish between whether the changes they are seeing in the bRGC population are due to loss of FOXP1 in the bRGCs or its loss in the aRGC population that they arose from. For instance, the increased IPCs reported in FOXP1-/- organoids could be as a result of early generation from the aRGC population. Numbers of aRGCs in the absence of FOXP1 are not reported. Similarly, the neuronal population the authors analyze also express FOXP1. Therefore, the impact on this population reported could be due to changes in the progenitor population (both aRGCs and bRGCs) or the neurons themselves.

We again agree with the reviewer that it is very challenging to disentangle whether our results in bRGCs are the results of effects of loss in aRGCs or bRGCs directly. However, we do not think our overall conclusions would be affected by this knowledge. For example, many studies report effects in neurons that are the consequence of gene manipulations that occur in earlier development (Park et al., 2022).

We now add additional results about aRGCs (see below and new Fig S6) and state in the manuscript:

“This early depletion of progenitors may have led to the observed reduction in neurons. Additionally, we observed a trend in the number of SOX2+ aRGCs being lower in the KOs compared to WTs, which denote the majority of aRGCs (Figure S6C), and we saw changes in the division angle at the basal side where aRGCs divide (Figure S6A). We observed that aRGCs at the basal side tend to divide more asymmetrically (oblique) with loss of FOXP1 compared to the WT, which is consistent with what had been shown in E13.5 mouse embryonic cortex (Pearson et al., 2020). Our interpretation of this result is that this increased asymmetric, oblique division may indicate division to generate two daughter cells of different cell fate such as IPCs or neurons (Chenn and McConnell, 1995; Uzquiano et al., 2018).”

“Importantly, our experiments cannot fully distinguish whether the observed alterations in bRGCs are due to effects solely in bRGCs or from loss of FOXP1 in aRGCs that give rise to bRGCs.”

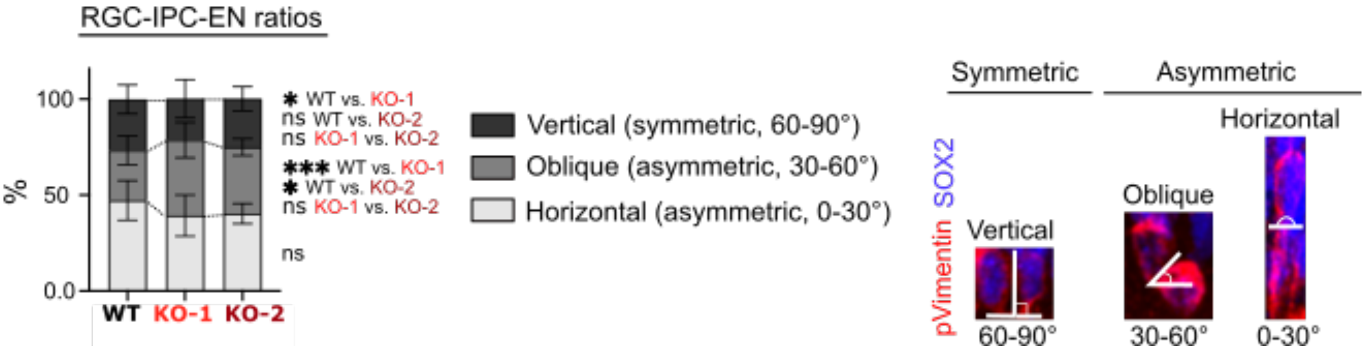

Representative images of symmetric and asymmetric division (right) and quantification of the divisions across WT, KO-1 and KO-2 (left).

* A more thorough analysis of cell populations in the absence of FOXP1 is required at different time points. Images should be improved, and interpretations should reflect the loss of FOXP1 in all cell types.

As mentioned above, we now include additional high-resolution images. We also temper our interpretations of our data with respect to the pseudotime analysis and the impact of FOXP1 loss on aRG versus bRG cells. It is not clear to us how many different time points would satisfy the reviewer as we capture all the major cell types that express FOXP1 by using Day 40 organoids.

We chose D40 because based on the cell type specific markers as well as FOXP1 expression, D40 reflected a small window of developmental stage when FOXP1 expression is the highest in the progenitors, bRGCs started to appear, and most cells and cellular layers are locally generated without subcortical inputs. Getting a snapshot of what the evolutionarily ancient gene regulator, FOXP1, is doing in an evolutionarily relevant cell type, bRGCs, at this specific time point was very important to us for those reasons.

We did try to see if we could examine bRGCs at later time points as well. Our D100 organoids show growth patterns similar to late second trimester or early third trimester human brain, such as expression of upper-layer specific markers (e.g. CUX1 and SATB2) relative to expression of lower-layer specific markers (e.g. TBR1 and CTIP2), a SVZ-specific marker (e.g. TBR2) and a VZ-specific marker (e.g. SOX2). However, the layers in the cortical plate were not as distinct as what can be observed in-vivo. One may attribute this problem to lack of circuitry as the cortical plate expands not only from the cells locally generated from VZ and SVZ but also from a lack of intact circuitry or blood vessels. This is true not only in our own system, but also noted as one of the key limitations of using brain organoids by others (Lancaster et al., 2014; Shou et al., 2020; Matsui et al., 2020). Also, because the direction of the processes is not organized, which is consistent with what we see in the literature (Qian et al., 2016), we could not reliably quantify the amount of HOPX based on location and the morphology in addition to the markers. Below is an example of what we saw in D100 organoids when we stained for HOPX, TBR2 and CTIP2.

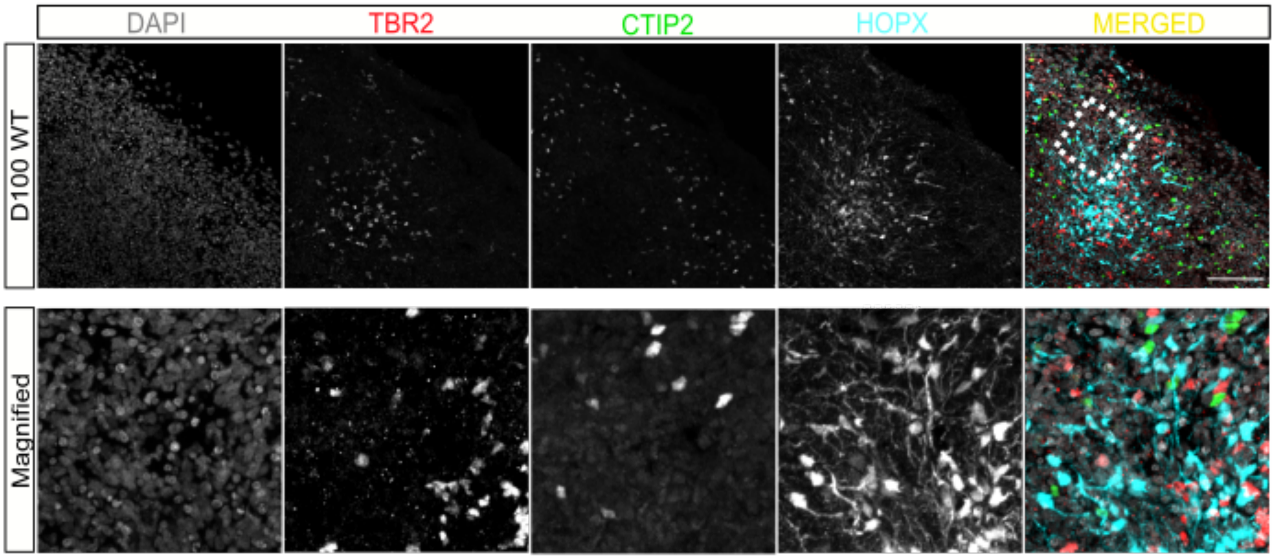

Representative staining from D100 organoids. Scale bar = 100µm

* I recognize that with an unconditional knock out it is not possible to determine the bRGC specific role of FOXP1. However, I do believe that there is value in reporting the loss of function phenotypes observed in human cortical organoids. These analyses can be improved and the conclusions re-interpreted to reflect the contribution of FOXP1+ aRGCs and neurons.

We again thank the reviewer for pointing out that we need to reassess the potential specific impact of FOXP1 on bRGCs compared to other cell types. We have improved our discussion to this end. We would also like to

point out that the number of DEGs in bRGCs is disproportionately high compared to the relative proportion of bRGCs there are. There is an order of magnitude fewer bRGCs compared to aRGCs or neurons in the organoids yet they are roughly 2/3 the number of DEGs in bRGCs as in aRGCs and more DEGs in bRGCs than in neurons. These results underscore the importance of FOXP1 as a transcriptional regulator in bRGCs. (see Figure 4A)

FOXP1 is an evolutionarily ancient protein important for human cognition and has a distinct expression pattern in bRGCs, a cell type linked to human brain evolution, in the early cortical development. Because it is a gene regulator that interacts with other gene regulators (either physically or through transcriptional regulation) and also regulates additional important development-related signaling pathways, we thought it would be crucial to have a snapshot of its activities in the actively developing early human brain model system.

With regards to the aRGCs and neurons, we do provide our results in Supplementary Figure S6C-E. Below is a summary of phenotypes we observed and DEGs potentially known to be linked to those phenotypes.

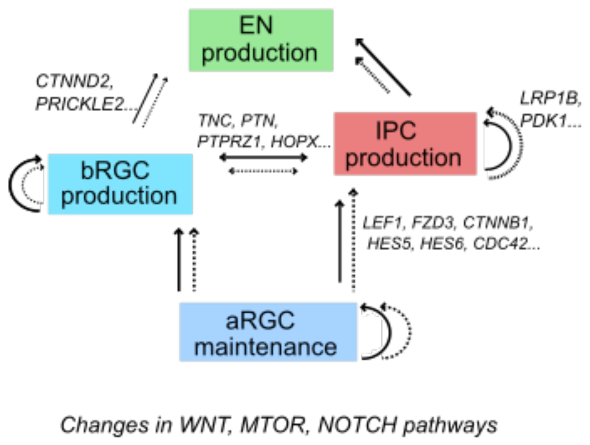

Because aRGCs and neurons are relatively well conserved between mouse and humans, the contribution of FOXP1+ aRGCs and neurons to cortical development have been examined in mouse models. In our laboratory, we are also actively investigating the role of FOXP1 in those cell types using conditional as well as inducible FOXP1 KO mouse models. It would be interesting to carry out comparative analysis of different aRGC and neuronal subtypes at single cell resolution and determine what role FOXP1+ bRGCs play in aRGCs and neurons that gave rise to or derived from FOXP1+ bRGCs, respectively. Perhaps carrying out a lineage tracing analysis paired with scRNA-seq would be an ideal experiment in the future in a separate study.

Although we did not describe in this manuscript directly evolutionary-relevant results, studying the role of FOXP1 in bRGCs specifically yielded results that could be potentially important in understanding the human specific features of bRGCs. We examined whether FOXP1 regulates expression of human/ape-specific genes in early cortical cells. We carried out enrichment analysis of a list of human/ape-specific genes with our DEGs and observed the most enrichment of these genes in bRGCs. Among the DEGs, *FAM182B* was most highly enriched in bRGCs. The gene encoding FAM182B is present in apes (i.e., chimpanzee, bonobo, gorilla and gibbon) but the protein is only expressed in humans due to the removal of a stop codon in the human genome (Florio et al., 2018). Previous work showed that *FAM182B* is highly enriched in cortical neural progenitors in the VZ and SVZ of human neocortex at gestation week (GW) 11 (Florio et al., 2018). Additionally, *GTF2H2C* is also a human-specific gene enriched in the cortical progenitors. Its function is not known. Along with *FAM182B*, we also detected *GTF2H2C* differentially regulated in bRGCs. Interestingly, we also found significant downregulation of *FOXP2* with loss of FOXP1 in bRGC cluster #25, IPC cluster#29, and six EN clusters (#5, 10, 15, 20, 21, 23, and 28). *FOXP2* is linked to human speech and language and ASD and is known to heterodimerize with FOXP1 (Li et al., 2004; Shu et al., 2007). Because these results are interesting yet challenging to definitively follow up without further experiments in organoids or tissue from multiple species, we did not discuss them in the manuscript.

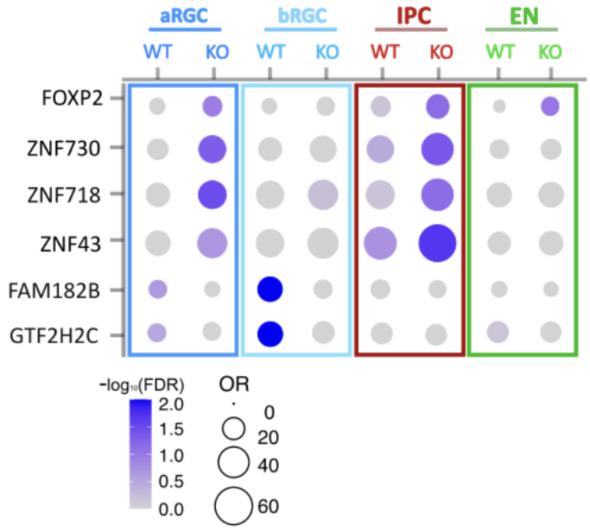

We now state in the manuscript:

"Because bRGCs are less common in rodent brain and do not express FOXP1 (Pearson et al., 2020), we decided to focus on bRGCs with respect to FOXP1 manipulation taking advantage of the relatively high percentage of bRGCs that express FOXP1 in the human brain organoids (>75%). Although bRGCs are a relatively rare cell type within the organoid model at D40 (~4%), use of the D40 timepoint allows us to capture the earliest role for FOXP1 in directing bRGC function when cortical lamination is still very clearly articulated."

References:

Andrews, M. G., Subramanian, L., & Kriegstein, A. R. (2020) mTOR signaling regulates the morphology and migration of outer radial glia in developing human cortex *eLife* 9:e58737.

Andrews, M.G., Subramanian, L., Salma, J., and Kriegstein, A.R. (2022). How mechanisms of stem cell polarity shape the human cerebral cortex. Nat Rev Neurosci *23*, 711-724. 10.1038/s41583-022-00631-3.

Arikkath, J., Peng, I. F., Ng, Y. G., Israely, I., Liu, X., Ullian, E. M., & Reichardt, L. F. (2009). Delta-catenin regulates spine and synapse morphogenesis and function in hippocampal neurons during development. *The Journal of neuroscience : the official journal of the Society for Neuroscience, 29*(17), 5435- 5442.

Bershteyn, M., Nowakowski, T.J ., Pollen, A. A., Di Lullo, E,. Nene, A., Wynshaw-Boris ,A., & Kriegstein, A. R. (2017) Human iPSC-Derived Cerebral Organoids Model Cellular Features of Lissencephaly and Reveal Prolonged Mitosis of Outer Radial Glia. *Cell Stem Cell*. 20(4):435-449.e4.

Braccioli, L., Vervoort, S. J., Adolfs, Y., Heijnen, C. J., Basak, O., Pasterkamp, R. J., Nijboer, C. H., & Coffer, P. J. (2017). FOXP1 Promotes Embryonic Neural Stem Cell Differentiation by Repressing Jagged1 Expression. *Stem Cell Reports, 9*(5), 1530 - 1545.

Chenn, A., & McConnell, S.K. (1995) Cleavage orientation and the asymmetric inheritance of Notch1 immunoreactivity in mammalian neurogenesis. *Cell*. 1995 Aug 25;82(4):631-41.

Close, J. L., Long, B. R., & Zeng, H. (2021). Spatially resolved transcriptomics in neuroscience. *Nature Methods, 18*(1), 23-25.

Cornell, B., & Toyo-oka, K. (2017). 14-3-3 Proteins in Brain Development: Neurogenesis, Neuronal Migration and Neuromorphogenesis. *Frontiers in Molecular Neuroscience, 10*, 318.

Desai, A. R., & McConnell, S. K. (2000). Progressive restriction in fate potential by neural progenitors during cerebral cortical development. *Development, 127*(13), 2863-2872.

Di Bella, D. J., Habibi, E., Stickels, R. R., Scalia, G., Brown, J., Yadollahpour, P., Yang, S. M., Abbate, C., Biancalani, T., Macosko, E. Z., Chen, F., Regev, A., Arlotta, P. (2021) Molecular logic of cellular diversification in the mouse cerebral cortex. *Nature.* 595(7868):554-559.

Eze, U. C., Bhaduri, A., Haeussler, M., Nowakowski, T. J., & Kriegstein, A. R. (2021). Single-cell atlas of early human brain development highlights heterogeneity of human neuroepithelial cells and early radial glia. *Nature Neuroscience, 24*(4), 584-594.

Florio, M., Heide, M., Pinson, A., Brandl, H., Albert, M., Winkler, S., Wimberger, P., Huttner, W. B., & Hiller, M. (2018, 2018/03/21). Evolution and cell-type specificity of human-specific genes preferentially expressed in progenitors of fetal neocortex. *eLife, 7*, e32332.

Florio, M., & Huttner, W.B. (2014) Neural progenitors, neurogenesis and the evolution of the neocortex. *Development*, 141(11): 2182-94.

Kaplan, E. S., Ramos-Laguna, K. A., Mihalas, A. B., Daza, R. A. M., & Hevner, R. F. (2017, 2017/08/16). Neocortical Sox9+ radial glia generate glutamatergic neurons for all layers, but lack discernible evidence of early laminar fate restriction. *Neural Development, 12*(1), 14.

Lancaster, M.A, & Knoblich, J.A. (2014) Generation of cerebral organoids from human pluripotent stem cells. *Nat Protoc.* 9(10):2329–40.

Li, S., Weidenfeld, J., & Morrisey, E. E. (2004, Jan). Transcriptional and DNA binding activity of the Foxp1/2/4 family is modulated by heterotypic and homotypic protein interactions. *Molecular and Cellular Biology, 24*(2), 809-822.

Li, Z., Tyler, W.A., Zeldich, E., Santpere Baró, G., Okamoto, M., Gao, T., Li, M., Sestan N. & Haydar T.F. (2020) Transcriptional priming as a conserved mechanism of lineage diversification in the developing mouse and human neocortex. *Sci. Adv.* 6: eabd2068.

Li, X., Xiao, J., Fröhlich, H., Tu, X., Li, L., Xu, Y., Cao, H., Qu, J., Rappold, G. A., & Chen, J.-G. (2015). Foxp1 regulates cortical radial migration and neuronal morphogenesis in developing cerebralcortex. *PLoS ONE, 10*(5), e0127671- e0127671.

Longo, S. K., Guo, M. G., Ji, A. L., & Khavari, P. A. (2021). Integrating single-cell and spatial transcriptomics to elucidate intercellular tissue dynamics. *Nature Reviews Genetics, 22*(10), 627-644.

Martinez, M. C., Ochiishi, T., Majewski, M., & Kosik, K. S. (2003). Dual regulation of neuronal morphogenesis by a δ-catenin–cortactin complex and Rho. *The Journal of Cell Biology, 162*(1), 99-111.

Martínez-Martínez, M.Á., De Juan Romero, C., Fernández, V., Cárdenas, A., Götz, M., & Borrell, V. (2016) A restricted period for formation of outer subventricular zone defined by Cdh1 and Trnp1 levels. *Nat Commun.* 7:11812.

Matsui ,T.K., Tsuru, Y., & Kuwako, K.I. (2020) Challenges in Modeling Human Neural Circuit Formation via Brain Organoid Technology. *Front Cell Neurosci.* 14:607399.

Matsumoto, N., Tanaka, S., Horiike, T., Shinmyo, Y., & Kawasaki, H. (2020) A discrete subtype of neural progenitor crucial for cortical folding in the gyrencephalic mammalian brain. *eLife* 9:e54873.

Noctor, S.C., Martínez-Cerdeño, V., Ivic, L., & Kriegstein, A. R. (2004) Cortical neurons arise in symmetric and asymmetric division zones and migrate through specific phases. *Nat Neurosci*. 7(2):136-44.

Nowakowski, T. J., Bhaduri, A., Pollen, A. A., Alvarado, B., Mostajo-Radji, M. A., Lullo, E. D., Haeussler, M., Sandoval-Espinosa, C., Liu, S. J., Velmeshev, D., Ounadjela, J. R., Shuga, J., Wang, X., Lim, D. A., West, J. A., Leyrat, A. A., Kent, W. J., & Kriegstein, A. R. (2017). Spatiotemporal gene expression trajectories reveal developmental hierarchies of the human cortex. *Science, 358*(6368), 1318 - 1323.

Nowakowski, Tomasz J., Pollen, Alex A., Sandoval-Espinosa, C., & Kriegstein, Arnold R. (2016). Transformation of the Radial Glia Scaffold Demarcates Two Stages of Human Cerebral Cortex Development. *Neuron, 91*(6), 1219-1227.

Park, S.H.E., Ortiz, A.K., & Konopka, G. (2022). Corticogenesis across species at single-cell resolution. *Developmental Neurobiology*, 82, 517– 532.

Pearson, C.A., Moore, D.M., Tucker, H.O., Dekker, J.D., Hu ,H., Miquelajáuregui, A., & Novitch, B.G. (2020) Foxp1 Regulates Neural Stem Cell Self-Renewal and Bias Toward Deep Layer Cortical Fates. *Cell Rep.* 30(6):1964-1981.

Pollen, A. A., Nowakowski, T. J., Chen, J., Retallack, H., Sandoval-Espinosa, C., Nicholas, C. R., Shuga, J., Liu, S. J., Oldham, M. C., Diaz, A., Lim, D. A., Leyrat, A. A., West, J. A., & Kriegstein, A. R. (2015). Molecular Identity of Human Outer Radial Glia during Cortical Development. *Cell, 163*(1), 55 - 67.

Qian, X., Nguyen, H.N., Song, M.M., Hadiono, C., Ogden, S.C., Hammack, C., Yao, B., Hamersky, G.R., Jacob, F., Zhong, C., Yoon, K.J., Jeang, W., Lin, L., Li, Y., Thakor, J., Berg, D.A., Zhang, C., Kang, E., Chickering, M., Nauen, D., Ho, C.Y., Wen, Z., Christian, K.M., Shi, P.Y., Maher, B.J., Wu, H., Jin ,P., Tang, H., Song, H., & Ming, G.L. (2016) Brain-Region-Specific Organoids Using Mini-bioreactors for Modeling ZIKV Exposure. *Cell.* 165(5):1238-1254.

Rosário M, Schuster S, Jüttner R, Parthasarathy S, Tarabykin V, Birchmeier W. (2012) Neocortical dendritic complexity is controlled during development by NOMA-GAP-dependent inhibition of Cdc42 and activation of cofilin. *Genes Dev*. 26(15):1743-57.

Rousso DL, Pearson CA, Gaber ZB, Miquelajauregui A, Li S, Portera-Cailliau C, Morrisey EE, Novitch BG. (2012) Foxp-mediated suppression of N-cadherin regulates neuroepithelial character and progenitor maintenance in the CNS. *Neuron*. 74(2):314-30.

Sauerland, C., Menzies, B.R., Glatzle, M., Seeger, J., Renfree, M.B., & Fietz, S.A. (2018) The Basal Radial Glia Occurs in Marsupials and Underlies the Evolution of an Expanded Neocortex in Therian Mammals. *Cerebral Cortex*. 28(1):145–157.

Schütze, T.M., Bölicke, N., Sameith, K., & Albert, M. (2023) Profiling Cell Type-Specific Gene Regulatory Regions in Human Cortical Organoids. *Brain Organoid Research* (17-41).

Shou Y, Liang F, Xu S, Li X. (2020) The Application of Brain Organoids: From Neuronal Development to Neurological Diseases. *Front Cell Dev Biol*, 8:579659.

Shu, W., Lu, M.M., Zhang, Y., Tucker, P.W., Zhou, D., & Morrisey, E.E. (2007). Foxp2 and Foxp1 cooperatively regulate lung and esophagus development. *Development, 134*(10), 1991-2000.

Turner, T.N., Sharma, K., Oh, E.C., Liu, Y.P., Collins, R.L., Sosa, M.X., Auer, D.R., Brand, H., Sanders, S. J., Moreno-De-Luca, D., Pihur, V., Plona, T., Pike, K., Soppet, D.R., Smith, M.W., Cheung, S.W.,

Martin, C.L., State, M.W., Talkowski, M.E., Cook, E., Huganir, R., Katsanis, N., & Chakravarti, A. (2015). Loss of δ-catenin function in severe autism. *Nature, 520*(7545), 51-56.

Uzquiano, A., Gladwyn-Ng, I., Nguyen, L., Reiner, O., Götz, M., Matsuzaki, F. and Francis, F. (2018) Cortical progenitor biology: key features mediating proliferation versus differentiation. J*. Neurochem*., 146: 500-525.

Valenta, T., Hausmann, G., & Basler, K. (2012). The many faces and functions of β-catenin. *TheEMBO Journal, 31*(12), 2714-2736.

Yang, L., Li, Z., Liu, G., Li, X., & Yang, Z. (2022, 2022/01/01). Developmental Origins of Human Cortical Oligodendrocytes and Astrocytes. *Neuroscience Bulletin, 38*(1), 47-68.

Yuan, L., Seong, E., Beuscher, J.L., & Arikkath, J. (2015). δ-Catenin Regulates Spine Architecture via Cadherin and PDZ-dependent Interactions*. *Journal of Biological Chemistry, 290*(17), 10947-10957.

Zhang Y, Pak C, Han Y, Ahlenius H, Zhang Z, Chanda S, Marro S, Patzke C, Acuna C, Covy J, et al. (2013) Rapid single-step induction of functional neurons from human pluripotent stem cells. *Neuron.* 78:785–798.
