# Supplementary material for: FOXP1 orchestrates neurogenesis in human cortical basal radial glial cells
Source: PLoS Biol. 2023 Aug 4;21(8):e3001852. doi: 10.1371/journal.pbio.3001852 (PMC10431666; doi:10.1371/journal.pbio.3001852)
Supplement: Attachment — Submitted filename: Park_Reviewer_Response.pdf [file pbio.3001852.s018.pdf]

We are pleased by the positive response of the reviewers and editors about our revised manuscript.

Reviewer #2: The authors have appropriately addressed my questions and concerns, and therefore I think this manuscript should be accepted.

Thank you!
